# The economics of abortion and its links with stigma: A secondary analysis from a scoping review on the economics of abortion

**DOI:** 10.1371/journal.pone.0246238

**Published:** 2021-02-18

**Authors:** Brittany Moore, Cheri Poss, Ernestina Coast, Samantha R. Lattof, Yana van der Meulen Rodgers

**Affiliations:** 1 Ipas, Chapel Hill, North Carolina, United States of America; 2 Department of International Development, London School of Economics and Political Science, London, United Kingdom; 3 Department of Labor Studies and Employment Relations, Rutgers University, Piscataway, New Jersey, United States of America; 4 Department of Women’s and Gender Studies, Rutgers University, Piscataway, New Jersey, United States of America; University of North Texas Health Science Center, UNITED STATES

## Abstract

**Background:**

Although abortions are a common aspect of people’s reproductive lives, the economic implications of abortion and the stigmas that surround abortion are poorly understood. This article provides an analysis of secondary data from a scoping review on the economic impact of abortion to understand the intersections between stigma and economics outcomes at the microeconomic (i.e., abortion seekers and their households), mesoeconomic (i.e., communities and health systems), and macroeconomic (i.e., societies and nation states) levels.

**Methods and findings:**

We conducted a scoping review using the PRISMA extension for Scoping Reviews. Studies reporting on qualitative and/or quantitative data from any world region were considered. For inclusion, studies must have examined one of the following microeconomic, mesoeconomic, or macroeconomic outcomes: costs, benefits, impacts, and/or value of abortion-related care or abortion policies. Our searches yielded 19,653 items, of which 365 items were included in our final inventory. As a secondary outcome, every article in the final inventory was screened for abortion-related stigma, discrimination, and exclusion. One quarter (89/365) of the included studies contained information on stigma, though only 32 studies included stigma findings directly tied to economic outcomes. Studies most frequently reported stigma’s links with costs (n = 24), followed by economic impact (n = 11) and economic benefit (n = 1). Abortion stigma can prevent women from obtaining correct information about abortion services and laws, which can lead to unnecessary increases in costs of care and sizeable delays in care. Women who are unable to confide in and rely on their social support network are less likely to have adequate financial resources to access abortion.

**Conclusions:**

Abortion stigma has a clear impact on women seeking abortion or post-abortion care at each level. Programmatic interventions and policies should consider how stigma affects delays to care, access to accurate information, and available social and financial support, all of which have economic and health implications.

## Introduction

Abortion stigma is pervasive across the globe, which can ascribe a negative attribute to discredit individuals who are associated with abortion [1, 2]. Although abortion stigma exists globally, it should be understood as a social process that is constructed and perpetuated within the dynamics of a specific local context [1–3]. Stigma related to abortion is perceived and internalized by individuals, and it is enacted and reinforced by social processes across all levels of the socio-ecology model [1, 4, 5]. Abortion stigma can be experienced by individuals seeking abortion care services, providers of abortion care, and others engaged in abortion care and access [1, 2].

Perceived and internalized stigma appears to be a common experience among people seeking care. Studies examining stigma at the individual (micro) level have found varying levels of perceived stigma among people seeking abortion care, which can manifest from interpersonal relationships and at the broader community, institutional, and societal levels. Stigma can also be associated with feelings of guilt and shame around seeking and/or receiving abortion care services [6–10]. Another phenomenon associated with stigma is the felt need to keep the abortion secret, which can contribute to social isolation and psychological distress [5, 7, 8, 10, 11]. Findings from studies focused on community level stigma confirm that abortion stigma and negative public perception of people seeking care is common. Studies in Mexico, Ghana, and Zambia found the majority of respondents had stigmatizing attitudes or beliefs towards women seeking abortions [9, 12, 13]. One study found that internalized stigma was higher among women who had grown up with strong negative norms about abortion in their families or communities [5]. Stigma is also found to be higher among religious communities and where abortion laws are more restricted [5, 6].

Abortion stigma has implications for both health care providers and women seeking health care services. A qualitative study that looked at the presence and intensity of abortion across five countries (Mexico, Nigeria, Pakistan, Peru and the United States) found that stigma is associated with a greater emotional cost when obtaining abortion services, and frequently results in women seeking services without their typical social support network [6]. Community-based stigma may contribute to delays in accessing safe abortion care as well as the choice to use unsafe methods to terminate a pregnancy [14, 15]. Stigma among abortion care providers can also impact access to care. Abortion providers can both report feelings of being marginalized or devalued for their work and/or they may also perpetuate stigmatizing attitudes which can influence women’s trajectory to information and care [14, 16]. Stigma towards abortion within the health care system has reduced access to care and siloed abortion services from broader reproductive health care [17].

Socio-legal dynamics also contribute to an environment of abortion stigma. Restrictive abortion legislation can both result from abortion stigma and perpetuate that stigma throughout society. Current societal social norms can hold a powerful influence over a nation’s laws and policies [1]. As a result, pervasive abortion stigma can lead to restrictive abortion legislation, thereby embedding and perpetuating this stigma within the core governing structure of the nation. The state can exert control over abortion and an individual’s reproductive rights through legal restrictions or criminalization of abortion and by failing to invest in making services accessible [1, 18].

Abortion laws, ideologies, and practices are closely intertwined with stigmas based on the view that people, particularly women, seeking abortion are straying from the predominant feminine ideals of society. This belief is reinforced through stigmatizing discourse, discrimination, and stereotyping at multiple levels of society [1]. Although abortion stigma is important to understand as it relates to an individual’s experiences and access to care, there is also a need for additional research to fully articulate the broader ways in which stigma affects individual access to care, the social environment for those seeking care, and policies and laws related to abortion access [9]. In particular, little attention has been paid to the economic implications of abortion stigma, and the costs and economic impacts that individuals may experience from a range of issues stemming from abortion stigma such delays or lack of access to care and discrimination and social exclusion.

To address this gap, this article provides a secondary analysis of the existing evidence identified in a scoping review on the economics of abortion services (including un/safe abortion and post-abortion care) and abortion policies. Results from the microeconomic, mesoeconomic, and macroeconomic analyses are presented in separate companion articles [19–21]. Our objective in this article is to provide key findings on the implications of abortion stigma and its links with microeconomic, mesoeconomic, and macroeconomic outcomes. To achieve this objective, the scoping review answers the following question: What are the economic costs and impacts of abortion-related stigma, discrimination, and exclusion?

## Methods

We took a systematic approach to finding evidence on the economics of abortion by conducting a scoping review of relevant literature. Following the Preferred Reporting Items for Systematic reviews and Meta-Analyses (PRISMA) extension for Scoping Reviews (PRISMA-ScR) and reporting guidelines [22], we developed a protocol [23] to ensure our review was manageable, transparent, and reproducible. We decided to conduct a scoping review instead of a systematic review because we were interested in analyzing the available, known evidence on the economic consequences of abortion care and abortion policies, and we expected varied evidence on this topic [24].

The scoping review considered any peer-reviewed journal article on induced abortion and/or post-abortion care from any world region (Table 1). Items also must have been published in English, French, Spanish, Dutch, or German from 1 September 1994 to 15 January 2019. Additionally, the articles must have qualitative and/or quantitative data on one of the following economic outcomes of abortion care or abortion policies at the microeconomic, mesoeconomic, and/or macroeconomic levels: costs, impacts, benefits, and value. Through testing these search terms, we found these outcome categories to be broad enough to capture the many various terms related to economic outcomes while also capturing the documented economic consequences of abortions at the individual, community, and health systems levels.

**Table 1:** PICOTS criteria used in the scoping review.

PICOTS	Micro-level	Meso-level	Macro-level
Populations	Girls and women who obtained abortions or post-abortion care and members of their households	Communities and health systems in which girls and women obtain abortions or post-abortion care	Societies and nation states in which girls and women obtain abortions or post-abortion care
Interventions	Induced abortion (safe/unsafe), post-abortion care, and/or abortion policies		
Control	None		
Outcomes	Quantitative or qualitative data on:		
	economic costs of abortion care or abortion policies		
	economic impacts of abortion care or abortion policies		
	economic benefits of abortion care or abortion policies		
	economic value of abortion care or abortion policies		
Timeframe	1 September 1994 to 15 January 2019		

We chose eight electronic databases for searching: Cumulative Index to Nursing and Allied Health (CINAHL); EconLit; Excerpta Medica Database (EMBASE); International Bibliography of the Social Sciences (IBSS); JSTOR; PubMed; ScienceDirect; and Web of Science. These sources were searched using the tested, relevant search terms found in Table 2. These findings were supplemented with expert-recommended articles. We made no assessment on the quality of each item, as the purpose of this scoping review was to describe and synthesize the evidence base.

**Table 2:** Search terms and their combinations.

1. Abortion terms	2. Economic terms	3. Impact terms
abort*	cost*	cost*
termination of pregnancy	econom*	benefit*
terminate pregnancy	price*	value*
pregnancy termination	financ*	impact*
pregnancy terminations	resource*	
postabortion	fee*	
post-abortion	tax*	
	expenditure*	
	GDP	
	gross domestic product	
	pay*	
	expens*	

We used a data extraction template and codebook [23] to extract data into categories based on study background information, details of relevant economic outcomes, and information on our secondary outcome on abortion-related stigma, discrimination, and exclusion.

Given the preponderance of stigma associated with seeking and providing abortion services, we decided to screen every article in the final inventory for information on abortion-related stigma, discrimination, and exclusion as a secondary outcome. We categorized the stigma findings into the following levels based on where the stigma was perpetuated:

- At the micro-level, this would include internalized stigma as well as perceived and actual stigma experienced from family and close friends.
- At the meso-level, this would include provider and community-based stigma as well as stigma experienced within the local health system. This includes articles that discussed public stigma towards abortion providers.
- At the macro-level, this includes stigma perpetuated or institutionalized by societies and nation states.

While the decision-making framework above worked for many of the findings, we found that some results between the micro-, meso-, and macro-levels overlapped, as stigma can be experienced on a cross-cutting spectrum. For example, stigma can be experienced between an individual and their families, their community, and/or their health care system. For these cases, we included the findings in all relevant tables but only extracted higher-level key findings where themes emerged.

Findings in this article are reported using a systematic narrative synthesis framework in which the results are presented narratively and organized thematically, supplemented with tables of descriptive statistics on included studies and their outcomes.

### Methodological limitations

The results presented in this article are based on the secondary analysis of evidence identified in a scoping review on the economic costs and impact of abortion care and policies. As a voluminous body of research was found during the initial search, eight researchers (SRL, EC, YR, BM, CP, EZ, LG, JS) participated in the screening and data extraction stages of this work. To help ensure consistency across all researchers, common definitions for the primary economic outcomes and secondary outcomes were established, and a qualified process was developed, shared, and used across the team for data screening, application of the inclusion/exclusion criteria, and data extraction [23]. While these tools were used and the findings were checked, any shortcomings in the data collection and extraction remain a limitation of this scoping review process. To help minimize these limitations, JS conducted robustness checks and SL reviewed all extracted data for quality assurance.

Relevant material to this scoping review may have been missed due to the limitations in our inclusion/exclusion criteria, including grey literature, published literature outside of journals, and relevant literature published in languages other than English, French, Spanish, German, or Dutch.

### Findings

**Fig 1:**
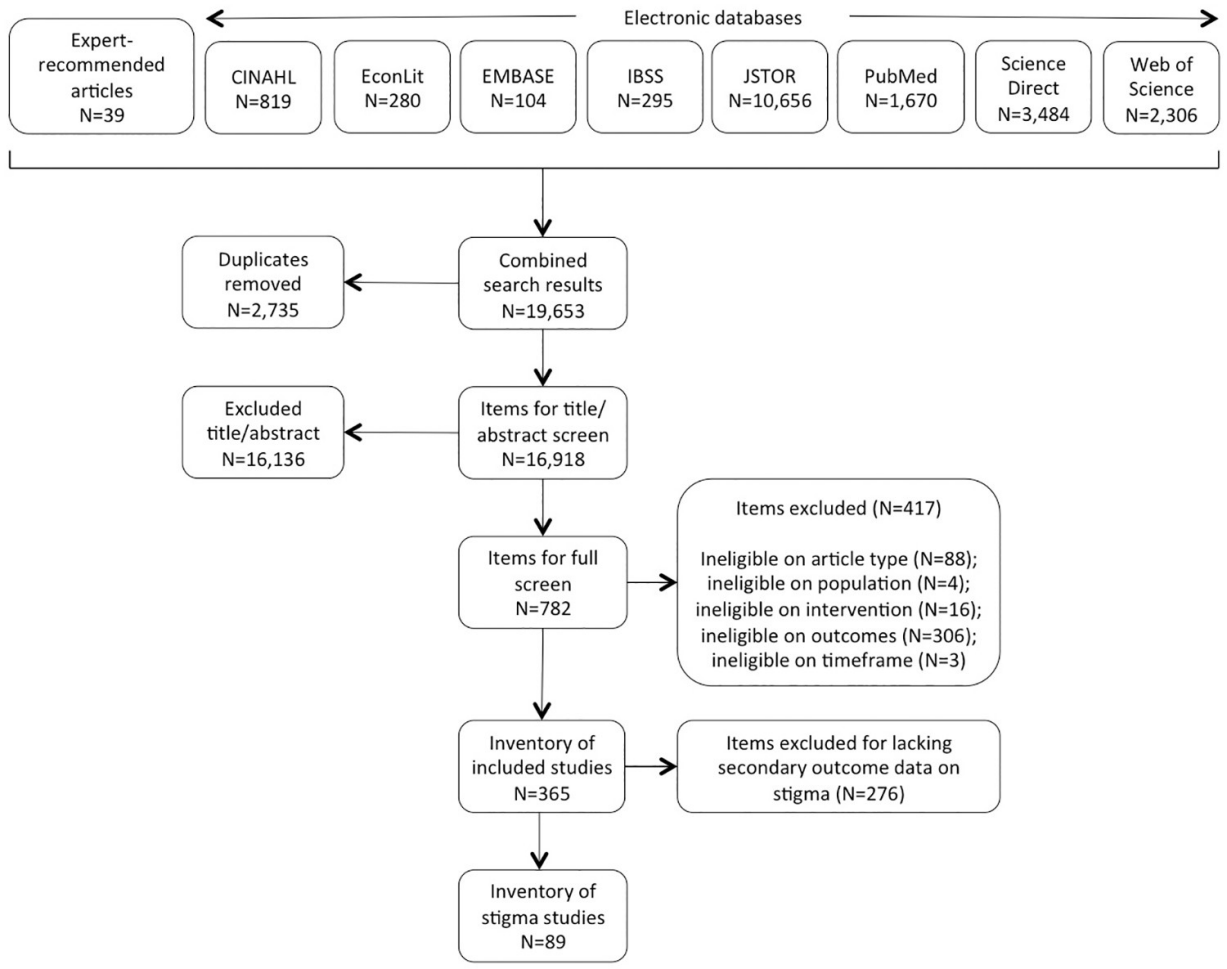
Screening results.

As shown in Fig 1, the search generated 19,653 items for screening. After duplicate removal, the 16,918 remaining items were screened for inclusion on the basis of title and abstract (TIAB). We determined eligibility of all items, and unclear items were discussed. Where exclusion could not be determined on the basis of TIAB, the authors screened the full text. Decisions were made in favor of an inclusive approach where questions remained. In total, 782 articles went through the process of a full text screening, where we screened on article type, intervention, outcomes, and timeframe. As a secondary outcome of the scoping review, we screened all articles in the final inventory of included studies (n = 365) for information on abortion-related stigma, discrimination, and exclusion. One quarter (89 out of 365) of the included studies contained information on stigma (Fig 1).

Based on the decision-making framework previously described in the methodology, over half of these studies examined stigma at the micro-level (51/89), followed closely by the meso-level (45/89) (Table 3). Only 27 articles referenced stigma at the macro-level.

**Table 3:** Level and content category of total included stigma studies.

Studies including stigma					
*Study reported on secondary outcome*		*# of studies by level*		*Total extractions by level*	
Yes	89	Micro	29	Micro	51
No	277	Meso	21	Meso	45
Total	365	Macro	11	Macro	27
		More than one	28	Total	123
		Total	89		

Note: Total data extractions by level include data extractions for studies with multiple levels.

As a secondary outcome, the related stigma findings within the 89 included articles ranged in direct relevance to the primary objective of this article. In order to provide key findings on the implications of stigma and its links with microeconomic, mesoeconomic, and macroeconomic outcomes, we focused on the articles that included stigma findings with direct economic links. The 89 included stigma articles were further examined to identify any connections to the primary economic outcomes of the scoping review.

Articles were organized into tables based on whether the stigma-based findings related to context, methodology, non-economic outcomes, or the primary economic outcomes (Table 4). Findings that referred to stigma within the general setting of the study were labeled as context (S1 Appendix). Two articles highlighted how stigma might have played a role in their data collection and were categorized as methodology (S2 Appendix). Articles that included stigma within their findings as key study outcomes but did not include related information regarding economic consequences were labeled as non-economic outcomes (S3 Appendix). From the 89 included stigma articles, only 32 included findings that were directly linked to the primary economic outcomes examined in the scoping review (S4–S10 Appendices).

**Table 4:** Content category of included stigma studies.

Content of stigma studies			
*Study reported on content category*		*Total # of findings by content category*	
Economic Outcomes	22	Economic Outcomes	32
Non-Economic Outcomes	44	Non-Economic Outcomes	54
Context	11	Context	11
Methods	2	Methodology	2
Economic and Non-Economic Outcomes	10	Total	99
Total	89		

Note: Total number of findings by category include studies with multiple content categories.

Of the 32 articles that included information on abortion-related stigma and links to economic outcomes, a majority focused on the United States (n = 9) (S1 Table). No other individual country or region comes close to the same number of studies conducted within the United States.

The majority of the studies utilized qualitative methods, with 16 studies relying exclusively on qualitative methods and 12 studies including both quantitative and qualitative methods (S2 Table). Half of the studies occurred in high-income countries (50%). A majority of the study intervention locations occurred at the sub-national level (56.3%), taking place within states or cities. The main identifier for the study population was more varied, ranging from geographical location (21.9%), status as abortion seeker (21.9%), abortion provider (12.5%), or a mix of multiple key identifiers (31.3%).

The 32 stigma articles that include links to the economic outcomes were then further organized into categories based on the economic outcomes of this scoping review (economic cost, impact, value & benefit). Ultimately, most of these articles included findings on economic cost (24/32) (S4, S7 and S9 Appendices), indicating that the field is aware of at least a general relationship between abortion stigma and economic cost. After economic cost, economic impact had the next highest reported findings (11/32) (S5 and S8 Appendices), and finally economic benefit (1/32) (S6 Appendix). The following sections focus on the key themes drawn from stigma findings related to the microeconomic, mesoeconomic, and macroeconomic outcomes (Table 5).

**Table 5:** Economic content and themes of included stigma studies (n = 34).

*Themes*	*n*
**Studies on microeconomic costs**	**12**
1. The ability to confide in a social support network has an impact on available financial resources to access abortion services	9
2. Abortion stigma can prevent people from accessing accurate information about abortion, which can lead to unnecessary increases in direct and indirect costs	3
**Studies on microeconomic impact**	**4**
1. Having a child without the financial means to support the child can lead to greater social stigma than choosing to have an abortion	3
2. Accessing abortion services can lead to loss of employment	1
**Studies on microeconomic benefits or value**	**1**
1. Women may act outside of their standard moral or religious values in order to advance their status in society	1
**Studies on mesoconomic costs**	**8**
1. Stigma from communities and health care providers can lead individuals to abortion care services outside the formal sector, which can have a meaningful impact of the cost of abortion services	5
2. Insurance companies can create a financial barrier to safe abortion services	3
**Studies on mesoeconomic impact**	**5**
1. Refusal to provide abortion services and/or referrals can result in substantial delays in care	4
2. Facility staff may provide inadequate information to individuals regarding public funding for abortion services	1
**Studies on macroconomic costs**	**4**
1. Anti-abortion movements and related political action restrict abortion access for women through legal regulations, which can result in increased financial barriers to care	4
**Studies on macroeconomic impact**	**2**
1. Monopolization of abortion services within the private sector has led to unequal access to services	1
2. The Global Gag Rule has institutionalized abortion stigma within its global foreign assistance structure	1

#### Microeconomic costs and consequences of abortion stigma

*Costs*. Of the 32 included studies with economic outcomes, nearly half examined the implications of stigma on economic cost at the micro level (15/323) (S3 Table; S4 Appendix). Studies on stigma and the costs of abortion at the micro level were conducted most often in the United States (n = 3) and Ireland (n = 2). One study examined micro-level costs from a global perspective and one article collected data at the regional level (Sub-Saharan Africa). The rest of the studies were conducted in individual countries (Bangladesh, India, Hong Kong, Cambodia, Australia, Kenya, Nepal). Evidence on the relationship between stigma and micro-economic cost outcomes came primarily from qualitative studies (n = 7) and data from mixed-methods studies (n = 4). The synthesis of this data has generated the following two themes regarding the associations between stigma and cost at the microeconomic level (Table 5).

*(1) People who are unable to confide in and rely on their social support network are less likely to have adequate financial resources to access abortion services* [25–33]. Fear of being stigmatized by a partner or family members could result in women reporting fewer financial resources and a lack of support in seeking abortion services [25, 26, 32, 33]. In these cases, women have had to make a difficult decision between their desire for confidentiality, the fear of being stigmatized by their social network, and having the financial resources to afford the costs of abortion services. In some cases, lack of financial resources and the fear of stigma from family and friends prevented some women from seeking abortion services entirely [29].

In India, seeking out private practitioners could offer young women the additional confidentiality some sought due to the social stigma surrounding abortion in their communities [30]. Although abortion is legal in India, some private providers take advantage of young women’s need for confidentiality and fear of social ostracism, charging three to five times the normal rate for abortion services. At this high cost, women can only feasibly access these services with financial support from their parents, brothers, or partner.

Similarly, abortion service fees in Indonesia are inflated and uncontrolled, particularly for unmarried women, because most abortion services are provided outside of the legal indications. While abortion is illegal except in cases to save the life of the woman, unmarried women are also unable to access maternal or family planning health services in general [28]. To avoid social condemnation, unmarried women seek out more confidential abortion services through private providers operating outside of the legal system.

The average cost of abortion services in Indonesia is beyond what most women can afford without the support of their partner, family, or friends [28]. The follow-up appointments are typically free, though women are usually not informed of this and many more miss these appointments due to the need for secrecy and to preserve their social reputation. Without the support from family or friends, women are less likely to attend the follow-up appointments, potentially leaving them at risk if they have any complications.

Conversely, women who could rely on their social support network for accurate information, facility recommendations, and financial support were more likely to be able to access and afford the costs of abortion services [27, 31]. In one study, women who received financial support from family members were able to visit private doctors in confidential settings with higher quality of care [27]. In addition to the direct cost of service, women’s social support networks were found to be essential to help minimize indirect costs associated with clinic visits, including transportation costs and childcare [26].

(2) *Abortion stigma can prevent individuals from obtaining correct information about abortion services and laws*, *which can lead to unnecessary increases in direct and indirect costs of care* [34–36]. Abortion stigma can make it challenging for women to find accurate and timely information about abortion services. Even in countries where abortion is legal, availability of information on abortion may vary throughout the country [35]. The desire for secrecy can also lead people to travel to clinics outside their communities for consultations [31]. Women may also feel uncomfortable asking for information due to the possibility of facing stigma from peers.

In some settings, women with low incomes can find it challenging to find affordable and accessible basic primary care services with qualified service providers [36]. These barriers can compound when seeking out providers for stigmatized services, like abortion or incomplete abortion care, as information around where to safely access these services can be challenging to find. Even in cases where care was subsidized, many low-income women were unaware of these services and were therefore not coming into facilities for consultations [36].

Delays in receiving accurate information on abortion services can result in unnecessary increases in direct and indirect costs of care. In Australia, one study participant reported traveling four hours for an abortion procedure despite living near a hospital that could have offered the same service [34]. Another study participant had to visit five general practitioners before she was able to receive a referral for abortion services, which delayed the timing of the procedure and resulted in compounded, unnecessary costs [34]. Reducing abortion stigma is necessary in order for women to receive accurate information in a timely manner and to reduce costs of service.

*Impact*. Only four articles included data on the economic impact of abortion stigma at the micro level (S3 Table; S5 Appendix); fewer than the previous section on economic cost and abortion stigma. These studies were conducted in individual countries (Indonesia, Ireland, Columbia, and Zambia). Data were primarily collected through qualitative methods (n = 3), though one study used a mixed-methods approach. Despite the limited evidence, the following two emerging themes highlight the potential economic impact of abortion stigma at the micro level (Table 5).

(1) *Having a child outside of marriage or without the financial means to raise the child can lead to greater social stigma than choosing to have an abortion* [28, 32, 37]. In Colombia, several study participants claimed that the social stigma attached to having a child in their current unmarried and financially unstable position would outweigh any stigma around having an abortion. The participants also explained that dropping out of school to raise the funds to support a new child would lead to judgment from their families [37]. In Ireland, a woman reported a doctor crying after she expressed a desire for abortion and trying to convince her that it was possible to raise a child while at college [32]. The stigma of having a child when not financially ready to do so was an important factor in the study participant’s decision-making.

Similarly, study participants in Indonesia said that while they strongly believed that abortion was a sin, it could be acceptable under certain specific circumstances [28]. In general, women tended to think that abortion was acceptable if marriage was not possible or if the man wanted to have an abortion. Many women also believed that abortion was necessary to avoid personal and family shame if the man refused to take responsibility of the child and get married, as having a child out of wedlock is perceived as a greater sin than abortion.

*(2) Accessing abortion services in a highly stigmatized environment can lead to loss of employment* [38]. In Zambia, stigma around receiving abortion services can have an adverse impact on a woman’s livelihood. One participant mentioned that she travelled to another town to receive abortion care services in an attempt to keep her abortion confidential. However, her employer found out about the procedure and she was ultimately fired from her job as a result [38].

*Benefits*. Only one of the 32 articles included findings on abortion stigma and economic benefits, and it presented evidence at the micro level (S3 Table; S6 Appendix). This study, conducted in Côte d’Ivoire, generated qualitative evidence on the relationship between abortion stigma and benefits. The main emerging theme from this study is: (1) *Women may act outside of their standard moral or religious values in order to advance their status in society* [39]. Study participants explained how abortion typically fell in strong opposition to their values and norms both in their community and as Muslims. However, they have begun to act outside of their standard moral and religious norms because of a shift in priorities. As Muslim women in Côte d’Ivoire are seeking access to education and financial independence in their communities, they found themselves advocating for abortion access as a means to achieve educational and financial goals for independence [39].

#### Mesoeconomic costs and consequences of abortion stigma

*Costs*. The implications of stigma on economic cost is the most frequently reported outcome at the meso level (10/32) (S3 Table; S7 Appendix). Studies were conducted most often in the United States (n = 3) while the rest of the studies were conducted in individual countries (Indonesia, Colombia, Poland, Zambia, Mexico, India, Kenya). Evidence on the relationship between stigma and meso-level cost outcomes came mostly from qualitative studies (n = 5). Synthesis of the evidence within these articles has generated the following two themes (Table 5).

(1) *Community and provider-based stigma around abortion can lead individuals to abortion care services outside of the formal sector or outside of legal restrictions*, *which can have a meaningful impact on the cost of abortion services* [28, 30, 40–42]. Pervasive community-based and perpetuated stigma can lead women to seek abortion services outside of the public sector, which can result in exorbitant, unregulated service fees. Providers outside of the formal sector can capitalize on abortion stigma, using it to charge unofficial and illegal fees to women who are in desperate need of confidential services [41].

In Poland, public-sector doctors refused to provide safe, affordable care in their facility because the doctors counted on women to “cope” with their abortion needs in the private sector [40]. Some of these doctors refused to provide care in the public sector but referred women to their own private practices. The private practice of abortion services is unregulated and untaxed, which can be an appealing source of income for some providers [40]. In one study in India, providers would refer to abortion services requested by unmarried adolescents as “illegal” services, even though these cases are legal under the abortion law in India [30]. Some providers mentioned that unmarried women were also charged, on average, three to five times the normal rate for an abortion.

As abortion services in Indonesia are only available to save the life of the woman, many women seek out services outside of the formal health sector. Additionally, unmarried women are largely unable to access any maternal health care services, so unmarried women in particular rely on confidential services to avoid social stigma and ostracization. However, confidentiality came at a high price, as providers charged between 60,000–300,000 rupiah for abortion services [28]. This cost usually covers the fee for the service, but does not include the cost of necessary drugs (i.e., pain management pills). Unmarried women interviewed mentioned that prices within this range are typically more than a woman could afford on her own, while one respondent said that her abortion fee was equal to two months of her boyfriend’s wages. The high, unregulated costs charged by providers outside of the formal sector represent a substantial barrier to women accessing safe abortion services. Many women interviewed stated that they delayed contacting a qualified provider due to the inaccessible cost of the service.

One participant from a study in Kenya said that some public-sector providers are willing to provide abortion services, but they only perform those services in their own private practice and for a considerable sum of money, noting that the cost of the service tends to depend on the demand for the service and whether the patient appears to have the means to pay [42]. As long as unofficial payments are made outside of the formal health sector, it will continue to be challenging for women to know how much an abortion should cost as well as their legal right to obtain that service within the formal sector [41].

(2) *Insurance companies can create a financial barrier to safe abortion services* [37]. One study in Colombia found that insurance companies, and more specifically company representatives, acted as a barrier to safe, affordable abortion care [37]. This study found that some company representatives refused to provide accurate information about coverage for abortion services, even though they were legally obligated to both provide the information and cover the procedure. The representatives were religiously motivated to not provide abortion service information [37].

*Impact*. Four articles included data on the economic impact of abortion stigma at the meso level (S3 Table; S8 Appendix). A majority of the articles took place in high-income countries (Canada, United Kingdom, United States). Evidence on economic impact of abortion stigma at the meso level was collected through qualitative studies (n = 3) and a cross-sectional survey (n = 1). Despite the relatively limited evidence base, data from these articles have been synthesized to develop the following two emerging themes that highlight the potential economic impact of abortion stigma at the meso level (Table 5).

(1) *Refusal to provide abortion services and/or referrals can result in substantial delays in care* [43–46]. Providers who refuse to provide care or referrals, despite the legal availability of abortion, can lead to long delays in care. In a study in the United Kingdom, four women reported seeking abortion outside of their home country because of the procedural barriers to legal abortion care [44]. One of the greatest barriers was the obstruction of access to care by clinicians, many of whom refuse to provide care due to their own personal beliefs. In various settings where abortion services are legally permitted, some abortion providers reported harassment or faced stigma from their colleagues. For example, in Mexico City, obstructing providers and other healthcare professionals would create an atmosphere of hostility in the facility, resulting in longer wait times for abortion services [46].

A study participant in Canada explained how their doctor refused to provide care or refer to another facility because it was against the doctor’s beliefs [43]. Others participants in the United States were unable to get referrals from their doctors to receive abortion services in the hospital, which would have been covered by Medicare (the federal government health coverage program), because of the doctor’s personal beliefs [45]. Even in cases where providers do not hold personal views against abortion, perceptions of abortion opposition in the community can lead facility staff to withhold information about available services to women [45]. These findings point to the importance of continuing to investigate how refusals to provide abortion care are defined by providers and communicated to their patients [44], and to determine the overall economic impact on women and their decisions around care.

(2) Facility staff and *providers may provide inadequate information to individuals regarding public funding for abortion services* [45]. In the United States, confusion regarding state funding and rules regarding abortion can jeopardize that funding and lead to procedural barriers for women [45]. Facility staff may be reluctant to provide women with information about abortion out of fear that sharing information could threaten their state family planning funding. In an effort to protect their facility and livelihood, some providers may withhold critical, timely information to their patients regarding abortion-related care.

#### Macroeconomic costs and consequences of abortion stigma

*Costs*. As shown in S9 Appendix, four articles included data on the implications of stigma on economic cost at the macro-level (S3 Table). A majority of the included studies were conducted in high-income countries (United States, Poland, Ireland). Evidence on stigma and economic cost at the macro-level was collected through a mixed-methods study (n = 1), qualitative study (n = 1), literature review (n = 1), and regression analysis (n = 1). Data from the four articles have been synthesized to develop the following emerging theme (Table 5).

(1) *Anti-abortion movements and related political action restrict abortion access for individuals through legal regulations*, *which can result in increased financial barriers to care* [32, 40, 47, 48]. The individual decision to seek abortion services in the United States has become a topic of public discussion, as public opinion has the ability to define how easy, expensive, and safe it is to obtain an abortion [48]. The anti-abortion movement has been able to remove abortion care from other aspects of reproductive health and to shape public perception of abortion as a burdensome, immoral, and socially stigmatized act. Anti-abortion movements have also led movements to restrict and recriminalize abortion in order to restrict access for women and to reduce public funding for facilities that provide services [47]. Leading with an argument of moral values, anti-abortions movements have campaigned against public funding for abortion, claiming that people should not be forced to pay for something that is against their moral values.

As aggressive public backlash against abortion builds, more providers are unwilling to perform abortions because of the harassment and intimidation by the anti-abortion movement, the emotional stress of families in crisis, and the relatively low pay to compensate the risk [48]. As fewer providers are willing to take on abortion service provision, access to abortion services can continue to be more cost prohibitive for women who may need to visit multiple clinics and/or travel greater distances to seek care.

Due to the restrictive regulations in counties like Poland and Ireland, women must travel outside of their country in order to access abortion services [32, 40]. While women of higher socio-economic status have the potential means to travel, women in lower socio-economic classes are less likely to have the resources to do so. At the same time, those most affected by the restrictive regulations around abortion are unlikely to have the political and economic capital on their own to protest the law and enact change, trapping them in a discriminatory cycle.

*Impact*. Only three articles discussed the macroeconomic impact of abortion stigma (S3 Table; S10 Appendix). One of the articles examined economic impact at the global level, and the other two studies were conducted in high-income countries (United States and Poland). Evidence was gathered and presented through a mixed-methods study design (n = 1), a literature review (n = 1), and a regression analysis (n = 1). Synthesis of the data from these three articles has led to the development of the following two emerging themes (Table 5).

(1) *The monopolization of abortion services within the private sector has led to unequal access to services due to their unregulated and unsubsidized prices* [40]. Abortion stigma in Poland has successfully pushed safe, affordable services out of the public sector, which led to the development of a thriving, underground private sector market for abortion services [40]. Within the private sector, stigmatization of services ensures that the procedures and costs of services remain unregulated. This monopolization of abortion services in the private sector perpetuates social inequality because their services are out-of-reach to a portion of the population that is unable to afford the cost of service.
(2) *The Global Gag Rule has institutionalized abortion stigma within its global foreign assistance structure* [49]. The Global Gag Rule, a term referring to the United States policy that, in earlier iterations, restricted foreign assistance for reproductive health to organizations above that provide abortion services, information, or referral. The Global Gag Rule has successfully institutionalized abortion stigma within its global foreign assistance structure by enacting restrictions to available funding. Non-government organizations that receive funding from the United States are unable to use that funding for abortion services, referrals, or even to share information regarding abortion. They risk losing their funding if they do not follow these rules. With the most recent iteration of the Global Gag Rule, the restrictions have expanded.

## Conclusion

This article presented the key findings on the implications of abortion stigma and its links with microeconomic, mesoeconomic, and macroeconomic outcomes. While the scoping review uncovered a large number of articles with information on the economics of abortion, a smaller number of articles included links to abortion stigma. Review and synthesis of key data within the included stigma articles resulted in high-level findings, programmatic recommendations, and key next steps.

Delays in seeking care is a consistent theme in this article and in the scoping review evidence base. A delay in finding care can have a major impact on the type of abortion care sought (i.e., formal/informal providers or using safe/unsafe abortion methods) and can impact the gestational age at which care is sought or reached. Efforts to create pathways to abortion care services need to prioritize reducing delays to help alleviate some of these constraints.

Control over accurate information on abortion services and the ability or means to access correct information on abortion care is also an important emerging finding of this article. In several settings, abortion services were legal and/or provided for free or reduced cost in various facility settings, yet this information was not widely known by the individuals seeking services. Based on the findings of the systematic review, programmatic interventions designed to provide financial support to individuals seeking abortion care services should consider the ways in which stigma may affect individuals’ ability to access those interventions and sources of information. Communities, local and national governments, and health facilities should be held accountable for providing accurate and timely information on abortion to support individuals seeking care. Community-based and civil society organizations, community health workers and volunteers, and other community members must be informed and empowered to uphold human rights regarding access to abortion services. They should be committed to holding individuals and organizations accountable when they limit access to those rights. Such information is crucial given the extent to which stigma can affect an individual’s care-seeking behaviors.

Several studies (n = 9) commented on the connection between social support and available financial resources to access abortion services. Individuals without access to a social support network are likely to have fewer funds available to access services, whereas those with strong social support networks are more likely to afford abortion services. Programs and policies need to be aware of these underlying dynamics and may need to design or link to additional support mechanisms (i.e., abortion funds, vouchers, etc.) and should ensure an equity lens for these types of interventions. Individuals seeking care need access to a social support network, ideally from a diverse range of family and community members as well as societal support at large. Programs working to expand access to care need to directly address the issues of stigma and social support at the community level to support an individual’s self-efficacy and ability to seek care. Social-behavior change approaches can be utilized to develop interventions to identify knowledge on and use of abortion services, including common beliefs and behaviors, as well as other key levers for change in this context. Interventions should engage community and youth partners and focus on key stakeholders, family members, and other critical members of the social support network to support access to information and safe abortion services.

In addition to the key points above, the following recommendations are made to consider for programmatic interventions:

- Advocacy for universal health coverage that includes abortion and other sexual and reproductive health services is a key measure to reduce the financial burden on individuals seeking abortion services. Comprehensive universal health coverage would ensure that all individuals and communities receive essential, quality health services, free of stigma and with respect to human rights.
- Decriminalizing abortion in countries where abortion is criminalized will not only improve access to safe abortion services, but it will also reduce stigma against individuals who seek abortion services.
- Relaxing regulations around medication abortions (non-surgical procedures), strengthening of protocols for assessing eligibility for medication abortion (including no-touch protocols), and improving networks needed to assist people with self-managed abortion so they can access services and manage complications with reduced stigma.
- Integrating safe abortion and contraceptive services into a full range of reproductive health services rather than keeping them in silos will go a long way to end the marginalization of individuals who seek these services.
- Permanently rescinding the Global Gag Rule and repealing the Helms Amendment would reduce the constraints around funding for abortion services and reproductive healthcare services. Repealing this counterproductive legislation would increase physical and financial access to abortion care services while also reducing stigma around abortion services at the macro-level.

To strengthen the key findings and emerging results in this article, the following recommendations are made for future research and related work:

- A disproportionate amount of the existing evidence utilized qualitative methods and is focused on the United States. To strengthen the evidence base, it is critical that more research is conducted in other countries so the data are reflective of the global population. Researchers should also consider a more diverse set of methods to help examine and quantify the economic impacts of abortion stigma and policies. There are available, validated stigma scales that look at the issue of abortion stigma from multiple perspectives (individual, community, and provider), and it may be interesting to pair those tools with these economic outcomes of interest.
- The results of this analysis have also shown that the connections between stigma and economic benefits/values are less defined and understood. More research can be done to examine this connection.
- Alongside the recommendation to bolster social support, more research can be completed and documented to identify the most effective programmatic approaches to increase social support and reduce abortion stigma as well as the overall relationship with cost of services.
- While the majority of the articles analyzed included findings on how stigma impacted the economic cost of abortion services and/or policies, more work is needed to intentionally map and quantify the connections between stigma and cost.
- Additional research is needed on how stigma affects the availability and costs of services, particularly in unregulated markets.
- Research is also necessary in order to investigate how refusals to provide abortion care are defined by providers and communicated to their patients [44], and to determine the overall economic impact on women and their decisions around care.
- It is a critical time to collect and analyze evidence on the economic impacts of restrictive US foreign policy worldwide. More research on the economic cost and impact, particularly of the Helms Amendment internationally is needed to be able to quantify the impact of the institutionalization of stigma through the United States’ aid structures.
- More research is needed to investigate how macro-level trends in abortion stigma affect availability, utilization, and costs at the meso- and micro-levels, as well as the costs implications of national-level legal restrictions and anti-abortion movements.

## Supporting information

S1 Appendix Summary of included studies reporting abortion-related stigma and context. (DOCX)

S2 Appendix Summary of included studies reporting abortion-related stigma and methodology. (DOCX)

S3 Appendix Summary of included studies reporting abortion-related stigma and non-economic outcomes. (DOCX)

S4 Appendix Summary of included studies reporting abortion-related stigma and economic costs at the microeconomic level. (DOCX)

S5 Appendix Summary of included studies reporting abortion-related stigma and economic impact at the microeconomic level. (DOCX)

S6 Appendix Summary of included studies reporting abortion-related stigma and economic benefits at the microeconomic level. (DOCX)

S7 Appendix Summary of included studies reporting abortion-related stigma and economic costs at the mesoeconomic level. (DOCX)

S8 Appendix Summary of included studies reporting abortion-related stigma and economic impact at the mesoeconomic level. (DOCX)

S9 Appendix Summary of included studies reporting abortion-related stigma and economic costs at the macroeconomic level. (DOCX)

S10 Appendix Summary of included studies reporting abortion-related stigma and economic impact at the macroeconomic level. (DOCX)

S1 Table Included studies by region and country. (DOCX)

S2 Table Characteristics of included studies. (DOCX)

S3 Table Economic content and level of extracted stigma studies. (DOCX)
