# Supplementary material for: The economics of abortion and its links with stigma: A secondary analysis from a scoping review on the economics of abortion
Source: PLoS One. 2021 Feb 18;16(2):e0246238. doi: 10.1371/journal.pone.0246238 (PMC7891754; doi:10.1371/journal.pone.0246238)
Supplement: S1 Appendix — (DOCX) [file pone.0246238.s001.docx]

**S1 Appendix. Summary** **of included studies reporting abortion-related stigma and context (n=11)**

| **Author, year [country]** | **Aim/objective(s)** | **Population** | **Study type** | **Level** | **Summary of main findings** |
| --- | --- | --- | --- | --- | --- |
| (Bloomer and O'Dowd 2014)[Ireland] | This paper considers abortion tourism in Ireland, both north and south, and how the moral conservatism present in both jurisdictions has impacted on attitudes and access to abortion | Literature on women seeking abortion services in restricted settings. | Literature review and supplemental expert interviews | Micro, Meso, and Macro | The negative stereotypes of women who access abortion mask the fact that many abortions are undertaken to preserve the health and well-being of family members including other children, and that many women who access abortion services continue with other pregnancies. Silence and fear of social ostracism stop other women speaking out to support those who have availed of abortion, and discrimination against those who have accessed it completes the stigma process. Those who provide abortion services are stigmatised by names such as ‘murderer’, which contributes to the exclusion of pregnancy termination as part of reproductive healthcare by equating it to a crime. The development of the idea of foetal personhood in which the notion that the foetus should be afforded the same rights as a person has increased over the past 10 years. Through the media, culture and art, abortion stigma has become embedded in popular discourse and equates qualities and autonomy of a baby with those of a foetus, which makes its destruction easy to portray as violent, unjust and morally wrong. As policy and law are reflections of the dominant ideology, abortion stigma can be found embedded in the core pillars of many societies. A recent study of abortion education in the UK found widespread evidence of bad practice in schools, including the provision of mis-information and distressing material to students. |
| (Coast, Norris et al. 2018) [Global] | To present a new conceptual framework for studying trajectories to obtaining abortion-related care. | n/a | Review | Meso; Macro | Conscientious objection to abortion may reflect stigma or violence providers themselves perceive or experience, and/or serve to further stigmatise abortion care-seeking. In contexts where abortion is stigmatised and/or illegal (or perceived to be illegal) in general or at advanced gestational age, women self-induce using household objects, traditional methods, and abortion medications. In some settings, anti-abortion protests outside abortion providers may affect abortion care-seeking by encouraging women to avoid providers where they may have to confront them. |
| (Ely, Hales et al. 2017a) [United States] | Use a trauma-informed lens to explore abortion-related hardships in a previously understudied group: patients in the United States who received ﬁnancial pledges from the National Network of Abortion Funds’ (NNAF) Tiller Memorial Fund, to pay for an unaffordable abortion. | Patients in the US who received financial pledges from NNAF to pay for an abortion (n=3,999). | Cross-sectional descriptive analysis | Micro; Macro | This study explored the occurrence of hardships by patients from various regions in the United States. The ﬁndings support that patients in the South and Midwest experience the greatest average number of hardships related to abortion and access to care. This ﬁnding is not surprising, considering the hostile abortion climate that is present in the Southeast, which the Midwest has recently begun to reﬂect as well. |
| (French, Anthony et al. 2016) [United States] | To assess the association of clinician referral with decision-to-abortion time. | English-speaking women aged 19 years and older presenting for an abortion for all indications at the three abortion clinics in Nebraska [n=263] | Cross-sectional survey | Micro; Meso | The small number of clinician referrals — 16% of which were inappropriate — is likely multifactorial in origin: poor clinician education about where to refer patients, clinician discomfort discussing abortion and a culture of silence among clinicians driven by fear of marginalization. Women in our study may have been unwilling to openly acknowledge that they were considering abortion due to stigma. |
| (Friedman, Saavedra-Avendaño et al. 2018) [Mexico] | To examine how a legislative discrepancy (legalized access in the city center vs. restricted access in the remainder of the muncipalities in the metropolitan area) affects access to public-sector abortion across the Mexico City Metropolitan Area. | Data from Mexico City’s publica abortion program and census for 75 municipalities. | Case-control | Micro; Macro | Stigma has been shown to be an important factor for women seeking abortion in Mexico, which may be more prevalent where abortion is illegal. It is possible that living where abortion is illegal is associated with increased experienced stigma, even when legal abortion is available in another part of the same city. For these reasons, women outside of the legal abortion zone may forgo accessing abortion services, or they may ﬁnd it more feasible to access abortion illegally or outside of a health facility, which can be safe or may expose them to additional medical and legal risks. |
| (Garcete and Winocur 2006) [Mexico] | To analyze different aspects that affect access to legal abortion in Mexico City and the characteristics that currently define such abortion services | Women who obtained or solicited legal abortion services between 2002-2006 | Analysis of survey data | Macro | 9.64% of the total of women who between 2002 and April 2006 requested an ILE (legal pregnancy interruption) received a negative response. However, this office does not resolve with precision, for example, what to do when the victim does not remember the exact date of the rape incident. Several studies of sexual violence confirm that 'forgetting' is a typical reaction, an unconscious mechanism of defense; but this is not understood by the Public Ministry. The belief that women are lying is recurrent in the discourse of people who decide the legality of an abortion due to rape. |
| (Messinger, Mahmud et al. 2017) [Bangladesh] | To investigate the knowledge, attitudes and practices regarding mHealth of both MR [menstrual regulation] clients and formal and informal sexual and reproductive healthcare providers in urban and rural low-income settlements in Bangladesh. | MY clients (n=24) and health service providers (n=24) | Qualitative | Micro | Given the stigma associated with MR, using formal mHealth services or using mobile phones informally to seek health care can help ensure women’s privacy regarding obtaining MR service information. |
| (Payne, Debbink et al. 2013) [Ghana] | To describe major barriers to widespread safe abortion in Ghana through interviews with Ghanaian physicians on the front lines of abortion provision | Ghanaian physicians known for their commitment to safe reproductive health services (n=4) | Qualitative: Open-ended interviews with key informants | Macro | Abortion is stigmatized in Ghana for reasons including the high value placed on motherhood and social sanctions against premarital sexual relationships. Importantly, influential Christian and Muslim communities serve as drivers of societal opinion on such matters. Conservative interpretations within both religious groups consider abortion immoral, though, a wide range of beliefs regarding the permissibility of abortion early in pregnancy exists. Nonetheless, strict religious interpretations and beliefs influence societal attitudes toward women seeking abortion. |
| (Sutton 2017) [Argentina] | Explore how several clandestine zones build women's bodies in vital ways to the sovereign power of the State, both in dictatorship and in democracy | Women in Argentina | Meta-analysis | Macro | This mandate [penalization of induced abortion] already marks a form of exclusion from full citizenship, an exception to the rule of what are the fundamental rights of the members of the political community. The pregnant body (or body that may become pregnant) would seem to exempt the person in question from full citizenship and from their human rights. |
| (Wilder 2000) [Isreal] | This study uses data from the 1974-75 Israel Fertility Survey and the 1987-88 Study of Fertility and Family Formation to examine the changing determinants of abortion among Jewish women in Israel. | Women of reproductive age in Isreal | Regression analysis | Macro | Although Jewish law condones the use of abortion when the foetus poses a serious physical or psychological threat to the well-being of the mother, the use of abortion as a method of birth control is proscribed. Previous research has documented an inverse association between religiosity and the acceptance of abortion, and highly religious Jewish women may be less likely to admit having had an abortion because of the social ban on the practice. |
| (Winikoff, Hassoun et al. 2011) [United States, France] | To examine the commercial, political, regulatory, and legislative history of the introduction of mifepristone / misoprostol in France and the United States. | Abortion providers and seekers | Review | Macro | The technological innovation around medical abortion in the United States would not have been possible without the willingness of French science and industry to engage in abortion research. The attitudes of the French government and public were crucial in allowing registration of an abortion technology without resort to the automatic exceptionalism that has characterized all aspects of abortion research, services, and discussion in the United States. |

Bloomer, F. and K. O'Dowd (2014). "Restricted access to abortion in the Republic of Ireland and Northern Ireland: exploring abortion tourism and barriers to legal reform." Culture, Health & Sexuality **16**(4): 366-380.

Coast, E., A. H. Norris, A. M. Moore and E. Freeman (2018). "Trajectories of women's abortion-related care: A conceptual framework." Social Science & Medicine **200**: 199-210.

Ely, G. E., T. Hales, D. L. Jackson, E. A. Bowen, E. Maguin and G. Hamilton (2017a). "A trauma-informed examination of the hardships experienced by abortion fund patients in the United States." Health Care for Women International **38**(11): 1133-1151.

French, V., R. Anthony, C. Souder, C. Geistkemper, E. Drey and J. Steinauer (2016). "Influence of clinician referral on Nebraska women's decision-to-abortion time." Contraception **93**(3): 236-243.

Friedman, J., B. Saavedra-Avendaño, R. Schiavon, L. Alexander, P. Sanhueza, R. Rios-Polanco, L. Garcia-Martinez and B. G. Darney (2018). "Quantifying disparities in access to public-sector abortion based on legislative differences within the Mexico City Metropolitan Area." Contraception.

Garcete, N. U. and M. Winocur (2006). "El acceso al aborto por violación en la Ciudad de México: limitaciones, oportunidades y desafíos." Debate Feminista **34**: 162-184.

Messinger, C. J., I. Mahmud, S. Kanan, Y. T. Jahangir, M. Sarker and S. F. Rashid (2017). "Utilization of mobile phones for accessing menstrual regulation services among low-income women in Bangladesh: a qualitative analysis." Reproductive Health **14**: 1-11.

Payne, C. M., M. P. Debbink, E. A. Steele, C. T. Buck, L. A. Martin, J. A. Hassinger and L. H. Harris (2013). "Why Women are dying from unsafe Abortion: Narratives of Ghanaian abortion providers." African Journal of Reproductive Health **17**(2): 118-128.

Sutton, B. (2017). "Zonas de clandestinidad y “nuda vida:”

Mujeres, cuerpo y aborto." Estudos Feministas **25**(2): 889-902.

Wilder, E. I. (2000). "Socioeconomic and Cultural Determinants of Abortion among Jewish Women in Israel." European Journal of Population / Revue Européenne de Démographie **16**(2): 133-162.

Winikoff, B., D. Hassoun and H. Bracken (2011). "Introduction and provision of medical abortion: a tale of two countries in which technology is necessary but not sufficient." Contraception **83**(4): 322-329.
