# Supplementary material for: The economics of abortion and its links with stigma: A secondary analysis from a scoping review on the economics of abortion
Source: PLoS One. 2021 Feb 18;16(2):e0246238. doi: 10.1371/journal.pone.0246238 (PMC7891754; doi:10.1371/journal.pone.0246238)
Supplement: S2 Appendix — (DOCX) [file pone.0246238.s002.docx]

**S2 Appendix. Summary** **of included studies reporting abortion-related stigma and methodology (n=2)**

| **Author, year [country]** | **Aim/objective(s)** | **Population** | **Study type** | **Level** | **Summary of main findings** |
| --- | --- | --- | --- | --- | --- |
| (Hendrickson, Fetters et al. 2016) [Zambia] | To examine sales practices, knowledge, and behavior of pharmacy workers regarding medical abortion in 2009 and 2011 in Zambia, where hostile and stigmatizing attitudes still result in high rates of unsafe abortion. | Pharmacy workers at government-certified pharmacies in the intervention areas (76 pharmacies in November 2009 and 80 in November 2011) | Descriptive cross-sectional design | Meso | The mystery-client methodology provides a rare opportunity to elicit information about behaviors related to a stigmatized procedure. However, this means that each individual pharmacy worker’s position, education, and previous exposure to medical abortion information are unknown to the mystery client, and therefore, the researchers. As part of the pilot study, many pharmacy workers in these pharmacies had participated in a 1-day training program on compassionate treatment for people seeking information about induced abortion, evidence-based regimens for medical abortion, and the provision of referrals to facility-based induced-abortion services. |
| (Schiavon, Collado et al. 2010) [Mexico] | To know whether factors [fear of staff attitudes, ignorance about the law, lack of information on where to access services, fear of breaches in confidentiality, burdensome requirements] were driving women in Mexico City to continue seeking private abortion services despite the availability of low-cost, safe, legal abortion services in the public sector. | Health providers working in lower middle-class neighbourhoods with small community health facilities [n=135 private sector physicians] | Descriptive | Micro; Macro | Since abortion is still highly stigmatized in Mexico and concerns about breaking the law may still exist, there may have been under-reporting on some of the questions, particularly in regard to services before the law changed. |

Hendrickson, C., T. Fetters, S. Mupeta, B. Vwallika, P. Djemo and K. Raisanen (2016). "Client–pharmacy worker interactions regarding medical abortion in Zambia in 2009 and 2011." International Journal of Gynecology & Obstetrics **132**(2): 214-218.

Schiavon, R., M. E. Collado, E. Troncoso, J. E. Soto Sánchez, G. O. Zorrilla and T. Palermo (2010). "Characteristics of private abortion services in Mexico City after legalization." Reproductive Health Matters **18**(36): 127-135.
