# Supplementary material for: The economics of abortion and its links with stigma: A secondary analysis from a scoping review on the economics of abortion
Source: PLoS One. 2021 Feb 18;16(2):e0246238. doi: 10.1371/journal.pone.0246238 (PMC7891754; doi:10.1371/journal.pone.0246238)
Supplement: S3 Appendix — (DOCX) [file pone.0246238.s003.docx]

**S3 Appendix. Summary** **of included studies reporting abortion-related stigma and non-economic outcomes (n=54)**

| **Author, year [country]** | **Aim/objective(s)** | **Population** | **Study type** | **Level** | **Summary of main findings** |
| --- | --- | --- | --- | --- | --- |
| (Agadjanian 2002) [Kazakhstan] | To analyse and compare trends in abortion and contraception, women’s attitudes toward abortion, and their perceptions of problems associated with abortion and contraception in Kazakhstan. | Kazakh women aged 15-49 | Cross-sectional descriptive survey | Macro | Russified Kazakhs and, especially, Europeans are markedly different from non-Russified Kazakhs: The former two groups are significantly less likely to disapprove of abortion outright. Overall, younger women, regardless of ethnicity, were significantly more likely to disapprove of abortion, which may attest to shifting attitudes in society in response to the government’s efforts to promote contraception. Notably, moral considerations concerning abortion, the only aspect in which abortion may be seen as radically different from—and even incompatible with—contraception, are of little importance to Kazakhstani women, despite increasing condemnation voiced primarily by Muslim and Orthodox clerics. If the discourse on the immorality of abortion is to have any effect on the generalized tolerance of abortion, its impact will likely differ across the ethnocultural groups. |
| (Aiken, Gomperts et al. 2017) [Ireland] | To document the experiences and characteristics of women seeking and completing at-home medical termination of pregnancy through online telemedicine in Ireland and Norther Ireland.^[[1]](#footnote-1)^ | Women resident in Republic of Ireland and Northern Ireland who requested at-home medication abortion through online telemedicine [n=5650] | Cross-sectional descriptive | Micro; Macro | Women spoke of the stigma attached to pregnancy in some social circumstances, the stigma surrounding termination of pregnancy (TOP), and the psychological suffering caused by having to continue a pregnancy they did not want or feel they could continue. Women commonly felt shame and isolation due to the stigma surrounding TOP. The findings of this study suggest that the most negative component of women’s experiences with at-home medical TOP is the need to maintain secrecy and silence because of the social stigma and a fear of prosecution engendered by its illegal status. |
| (Aiken, Guthrie et al. 2018) [United Kingdom] | To examine reasons for seeking abortion services outside the formal healthcare system in Great Britain, where abortion is legally available. | Women resident in England, Scotland, and Wales who requested at-home medication abortion through online telemedicine. [n=209] | Cross-sectional descriptive | Micro | Participants indicated that they were ashamed and embarrassed to return to a clinic for an abortion if they have been there previously for an abortion as they believe they will be judged for having another one. Other participants mentioned that they could not go to their local clinic because their town is small and a family member works there, and everyone would know they had an abortion. These are some reasons women access abortion services outside the health system, despite their availability within the formal system. |
| (Aiken, Johnson et al. 2018) [Ireland] | (1) to examine the factors affecting whether women in Ireland choose to access abortion by travelling or by using online telemedicine; and (2) to explore their experiences in accessing care through each pathway | Women (n=38) identified through three organisations: Women on Web, Abortion Support Network, For Reproductive Rights Against Oppression, Sexism and Austerity.  Criteria: aged over 18, had an abortion within 8 years of study, lived in Ireland at time of abortion, had travelled or used telemedicine to access abortion care. | Qualitative in-depth interviews | Meso; Macro | Several study participants expressed feelings of stigma from their providers and community when seeking abortion services. At times, this was caused by the legal restrictions on abortion services. |
| (Altshuler, Ojanen-Goldsmith et al. 2017) [United States] | To compare women's needs and preferences in abortion to those in birth  To examine ways in which women's needs and preferences in abortion care differ from intrapartum care | Women who had individually experienced both birth and abortion (n=20) | Semi-structured intensive interviews and a validated Individual Level Abortion Stigma scale (ILAS) assessment | Meso | This study used the Individual Level Abortion Stigma scale to measure the degree of personal stigma of the participants. In regards to abortion care, three elements were found to impact results: affirmation as moral decision-makers, ability to determine degree of presence during the abortion process, and provision of care in a discreet manner to avoid being judged by others for having an abortion. Many participants detailed interactions with their providers. The response of the providers had a distinct impact on the internalized stigma of the participant. |
| (Aniteye and Mayhew 2011) [Ghana] | To explore and understand the reasons why women terminate their pregnancies and their experiences of seeking services in order to know what and how to reform services to reduce unsafe abortion. | Women admitted to the hospitals with incomplete abortions (n=131) | Semi-structured hospital-based survey | Macro | In a country where Christian morals are strong the high proportion of respondents who were single is significant because the social stigma of bearing children out of wedlock is great. Reflecting this, about one third of respondents said they aborted because they were not married and two-thirds said they aborted because of socio-cultural pressures. |
| (Appiah-Agyekum, Sorkpor et al. 2015) [Ghana] | To explore the factors that are likely to influence abortion decisions among University students in Ghana and their knowledge and perceptions on abortion. | Randomly sampled students of the University of Ghana (n=142) | Qualitative: FGDs | Meso; Macro | The student participants of this study claimed that they were more likely to abort if they felt that the pregnancy will bring shame, disappointment, or resentment from their family. This study further suggests that although a good number of students support the existence of liberal laws, entrenched religious beliefs and doctrines as well as socio-cultural factors may explain the strong anti-abortion sentiments held by some students. While serving as a basis for their support for the banning of abortions, these same factors may account for their refusal to support or undergo abortions even if necessary to preserve their health and/or that of the unborn child, as well as their acceptance and indulgence in abortion stigma. |
| (Appiah-Agyekum 2018) [Ghana] | To explore the abortion experiences of Ghanaian university students | Female undergraduate students at the University of Ghana [n=32] | Qualitative cross-sectional descriptive | Meso | Students who had used the services of blue star facilities in Ghana did not patronize them for subsequent abortions nor recommend their use to their peers because of the unfavorable proximity, stigma attached to persons seen entering or leaving the premises of safe abortion providers, acquired knowledge on administering the drugs, over-bureaucratic administrative processes, waiting time, and cost of services. |
| (Baird 2015) [Australia] | To describe the context through which medical abortion has become available in Australia since 2013. | Australia | Narrative review | Meso; Macro | The stigma attached to being an abortion provider continues to be a major disincentive for general practitioners, especially in small rural communities. General practitioners have also worried that if they become identified as providers of medical abortion, their practice will be overwhelmed by requests for abortion. Some doctors also expect resistance to provide medical abortion from colleagues and in local communities. A New South Wales sexual and reproductive health referral service reported that in the first year after mifepristone became a subsidised medicine, when about one third of their callers who were seeking an abortion were wanting a medical abortion, some of the small number of general practitioners on their referral list “requested to be removed from our database”. |
| (Banerjee, Andersen et al. 2012) [India] | To compare women with induced abortions with women with spontaneous abortion. | Women of reproductive age who were seeking care for postabortion complications [n=344] at one of 10 hospitals. | Cross-sectional descriptive survey | Micro; Meso | Lack of social support, lack of knowledge about the legality of induced abortion, and abortion-related stigma influenced the type of provider women approached for abortion care. |
| (Brack, Rochat et al. 2017) [Colombia] | This study sought to identify the key barriers to legal abortion, and to explore the ways they may work separately and together to delay the receipt of high quality, legal abortion care. | Women who obtained a legal abortion in Bogotá (n=17). | Qualitative: in-depth interviews | Micro; Meso | For four of the study participants, stigma associated with abortion led to delays in decision making and in obtaining services. Of the nine study participants who had told their partner about the pregnancy, three felt pressured by their partner to carry the pregnancy to term and to get married. The partners of these three participants voiced religiously motivated antiabortion attitudes; the women stated that this dynamic resulted in delays in decision making and in obtaining services. |
| (Casas and Vivaldi 2014) [Chile] | To describe a study of the criminalisation of abortion as a human rights violation in Chile. | n/a | Review | Macro | The Public Defender acknowledged in a newspaper article that the abortion law has an unequal effect on women, especially the poor. He remarked on the cases of two adolescent girls whose experiences exemplify how medical confidentiality may be violated. They had been raped, yet they were found guilty of illegal abortion and stigmatised twice by the criminal justice system, instead of being protected as victims of sexual violence. |
| (Casas-Becerra 1997) [Chile] | To highlight the gender and poverty-related discrimination that poor women having abortions face in Chile, and how the law is used to undermine medical confidentiality . | Case review files | Review | Meso; Macro | Most of the cases of women who had abortions in our study (76 per cent) had been reported to the police by the public hospital where they had sought treatment for complications. Save for one, all the hospitals we came across in the files of the Santiago prison were publicly run facilities providing medical care to the poor and indigent. The remaining 24 per cent of the women were reported to the police by partners, relatives or employers (11 per cent) and some were found out by police by mere chance (4 per cent). Most of these were charged following a confession by the woman, or were identified at the hospital if they happened to have taken the woman there. |
| (Chevrette and Abenhaim 2015) [United States] | Assess whether US state-level policies regarding  abortion and sexual education are associated with different teen birth and teen abortion rates | National teen birth and teen abortion rates | Regression analysis | Micro | The results of this particular study show that the main determinants of teen births are cultural and dependent on family and peer values, which would agree with recent publications. Teenagers worldwide tend to go along with what is socially accepted by peers and family. |
| (Chunuan, Kosunvanna et al. 2012) [Thailand] | The purpose of this descriptive study of women from southern Thailand, who had undergone a recent abortion (spontaneous, therapeutic and unsafe), were to obtain data regarding: pregnancy history; number of abortions and cost of abortion related treatments; abortion complications, impacts and related health care services; reasons for having an unsafe abortion; and, circumstances related to an unsafe abortion. | Women that received abortion services from one of six government hospitals | Convenience sample and questionnaire | Macro | Study participants who experienced an unsafe abortion stated they preferred to terminate their pregnancy as soon as possible. No doubt this was because they did not want others to know about their pregnancies. Since Thai women are expected to preserve their virginity and display sexual correctness, women who get pregnant before marriage tend to be morally condemned. Furthermore, premarital and extramarital sex are frowned upon in the Thai culture and there is little sympathy for those who become pregnant out of wedlock, especially since it is believed women, but not men, can control their sexual desires. Thus, having an abortion often is associated with the woman having a lack of morals and virtue. |
| (Contreras, van Dijk et al. 2011) [Mexico] | To examine the experiences and opinions of health care professionals after the legalization of abortion in Mexico City in 2007 | 64 semi-structured interviews with obstetricians/gynaecologists, nurses, social workers, key decision makers at the Ministry of Health, and others | Qualitative study using semi-structured interviews | Meso | Health care professionals participating in the study reported that they were subjected to comments from colleagues that were perceived as stressful as upsetting. Colleagues of participants would berate participants with verbal abuse. At times, ill-effects of this discrimination would extend to patients of the study participants. |
| (Cook, de Kok et al. 2017) [Malawi] | To investigate factors contributing to the limited and declining use of MVA [manual vacuum aspiration] in Malawi | PAC providing health workers of different cadres (doctors, nurses and clinical officers), genders and levels of experience and seniority | Qualitative study using interviews and supplemental observation | Meso | Many health workers described finding PAC challenging due to their personal belief that induced abortion is immoral. These feelings were influenced by religious beliefs and prevailing community beliefs and norms. Participants explained that PAC is often only associated with induced abortions by the public and there tends to be negative attitudes towards these women, with people considering them ‘sinners’ or ‘criminals’; these words and views were often reflected by health workers. PAC has been promoted as the least contentious service for reducing unsafe abortions’ adverse consequences, but it cannot be separated from the socio-cultural, religious and legal status of abortion. Negative attitudes surrounding abortion may lead to delays; women who delay seeking care are at increased risk of more severe complications such as sepsis, resulting in being more likely to require further procedures in theatre rather than MVA as an outpatient. Negative attitudes may also result in providers’ negative attitudes towards providing PAC, low prioritization of services and low motivation to improve services. |
| (David and Baban 1996) [Romania] | Explore, through individual in-depth interviews, psychosocial antecedents and consequences of the Romanian pronatalist policies banning importation of contraception, prohibiting abortion, and imposing tax on childless couples. | Women seeking abortion services prior to the revolution (n=50). | Qualitative: open-ended interviews | Micro | Unmarried women who considered single parenthood a social stigma were in an especially difficult situation. |
| (de Bruyn 2003) [Global] | Summarises the results of a literature review on the subject of unwanted pregnancy and induced abortion among women living with HIV/AIDS. | Women living with HIV/AIDS | Review | Micro; Meso | In India in 2002, two nurses reported: ‘‘I was working as a nurse in a reputed Mumbai hospital and came to know about being HIV positive when I miscarried. I was bleeding profusely, but the gynaecologist refused to even touch me. I was shifted to a municipal hospital, but had a similar experience there. |
| (Diniz, d'Oliveira et al. 2012) [Brazil] | To examine equity in health and health care in Brazil, examining unjust disparities between women and men, and between women from different social strata, with a focus on services for contraception, abortion and pregnancy. | Brazil | Review | Meso | A recent study evaluated the quality of abortion care for women admitted to public hospitals in three state capitals. The care provided was far below the standards set by the Brazilian government, and pain management was frequently inappropriate. Sharp curettage (D&C) was the method used in almost all cases, which requires analgesia or deep sedation, hospital admission, longer waits for treatment, an overnight stay of at least 24 hours, and a greater risk of complications. Maintaining this outdated practice violates the principle of integrity and the aim of replacing less safe interventions with safer ones. The study also found other forms of discrimination, such as the postponement of curettage until night shifts. |
| (Dobie, Gober et al. 1998) [United States] | 31 family planning clinic sites in rural Washington State were surveyed about their sponsorship, stafﬁng, service provision and population coverage. | Family Planning clinics in rural Washington (n=31). | Cross-sectional survey | Meso | Study results indicated that the two most important reasons for not providing onsite abortions were local community opposition and lack of a trained provider. The two most common reasons rated as unimportant were the moral or religious concerns of staff and the availability of the service at another local facility. |
| (Doran and Nancarrow 2015) [United States] | To identify the factors that facilitate and hinder access to abortion services for women in developed countries in relation to first-trimester abortions. | n/a | Systematic Review | Meso | Actual or potential harassment influences hospital and provider willingness to provide abortions. One in five advanced clinicians identified fear of anti-abortion harassment as a perceived barrier to offering medical abortion. Several studies explored provider attitudes towards abortion and abortion law. As only 2/114 family physicians surveyed perform surgical abortions it was not surprising that 80% of physicians in this study had moral objections to abortion. Reasons for not providing abortion services were religious and community opposition. Negative attitudes of non-physician staff restricted access to abortion, and impacted women’s experiences of abortion services. Some GPs in Norway reported ambivalence towards their own refusal practices related to a non-absolutist conscientious objection stance illustrated by willingness to make certain compromises to refer women. Although most physicians surveyed in the USA did not report an objection to abortion in general, abortion for gender selection was not supported by 75% of participants. |
| (Doran and Hornibrook 2016) [Australia] | Identify factors that women in rural New South Wales (NSW) experience in accessing abortion services and suggestions about how rural women could be better supported when seeking access to an abortion service. | Women who sought access to abortion while living in a rural part of NSW (n=13). | Qualitative: in-depth interviews | Micro; Meso; Macro | An emergent theme was of women’s experiences of stigma, shame and secrecy. External stigma was exacerbated by protestors, and internalised stigma was linked to feelings of shame and secrecy. Some women discussed the consequences of stigma and the lack of respect for women to make reproductive decisions concerning their own bodies. Stigma and negative characteristics linked to abortion are perpetuated by social and political processes including media portrayals, which in turn influence women’s experiences, examples of which have been reported by women in this study. Women in this study point to non-integrated health systems, as evidenced by their experience of sub-standard primary care where doctors did not readily refer, did not provide accurate information about self-referral options and expressed judgemental attitudes reflective of their moral objections. |
| (Esia-Donkon, Darteh et al. 2015) [Ghana] | Explore the pre and post experiences of young people (aged 12 to 24) who had their abortion three months prior to the study. | Young people (12-14) who received abortion services at the Planned Parenthood Association of Ghana Cape Coast clinic (n=21). | Qualitative: in-depth interviews | Micro; Meso; Macro | With the perception that abortion was illegal, ‘murderous’ and sinful, the respondents generally found it difficult to take the decision even though to them, there was no other option available. Some reported that their sexual partners were against the abortion and therefore threatened a break-up of the relationship if the pregnancy was terminated. The respondents were aware of both public and private health facilities where abortion services are provided. However, apart from being a youth friendly centre, the choice of the PPAG clinic was based on other reasons. The location of the facility is convenient in that it does not attract many potential ‘gatekeepers’ and gossips, and the attitude of staff is youth friendly and compassionate. Psychologically, the respondents were worried about the fact that an ‘illegality’, a ‘crime’ and sin had been committed against humanity and God. This guilt led to self-imposed stigma, which was usually reinforced by sermons and talks about abortion at the Church, in the media and no other social platforms. |
| (Finer, Frohwirth et al. 2005) [United States] | Examine the steps in the process of obtaining abortions and women’s reported delays in order to help understand difficulties in accessing abortion services. | Women seeking abortion services (n=1,247). Location and facility details presented in another publication (Finer 2005). | Mixed methods | Micro | Although some described abortion as sinful and wrong, many of those same women, and others, described the indiscriminate bearing of children as a sin, and their abortion as “the right thing” and “a responsible choice.” Respondents often acknowledged the complexity of the decision, and described an intense and difficult process of deciding to have an abortion, which took into account the moral weight of their responsibilities to their families, themselves and children they might have in the future. |
| (Foster, LaRoche et al. 2017) [Canada] | To document women's experiences obtaining abortion care in New Brunswick (NB) before and after the Regulation 84-20 amendment; identify the economic and personal costs associated with obtaining abortion care; and examine the ways in which geography, age and language-minority status condition access to care. | New Brunswick residents who received abortion services (n=36). | Qualitative: semi-structured interviews | Micro; Meso | Referring a woman elsewhere to obtain information or a referral for the procedure compromises patient care, especially when the procedure is time sensitive. Referrals, let alone referrals for referrals, create significant delays in scheduling and further contribute to the burden faced by those women seeking services. |
| (Gerdts, Raifman et al. 2017) [South Africa] | To investigate the reasons for attempting self-induction, methods used, complications, and sources of information about informal sector abortion, and test a recruitment method. | Women [n=41] who sought informal sector abortion services in Cape Town, South Africa using respondent driven sampling (RDS). | Cross-sectional descriptive survey | Micro; Meso | 30% of women in our study cited seeking an informal sector abortion because of concerns about privacy, mistreatment and stigma from providers is a striking commentary on the perceptions of quality of abortion care in the formal sector. In a sample population consisting of many sex workers, mistrust of the formal health care system is not unexpected; sex workers seeking abortion in Cape Town likely face a double stigmatization related to abortion and their work. |
| (Glenton, Sorhaindo et al. 2017) [Bangladesh, Ethiopia, Nepal, South Africa and Uruguay] | To explore factors influencing the implementation of role expansion strategies for non-physician providers to include the delivery of abortion care | Non-physician providers of abortion services within multiple health facilities in each country non-physician providers | Qualitative: Case study, literature review, and key informants | Meso | In South Africa, some nurses, midwives, specialist and non-specialist doctors described feelings of rejection, stigma and negative comments because of their work from colleagues who did not provide abortion care, particularly when delivering second-trimester abortions. South African abortion providers were also reported as avoiding abortion training because of stigma from colleagues. |
| (Gober 1997) [United States] | This paper investigates the role of access in explaining the variation in state abortion rates. | States in the United States (US) | Regression/Path analysis | Macro | After the Supreme Court's refusal in 1992 to overturn Roe and eliminate the right for women to have abortions, militant antiabortion groups moved the battle over abortion rights from the courts to the streets. The National Abortion Federation estimated that incidents of violence (including bombings, arson, vandalism, death threats, and the stalking of clinic personnel) increased from 131 in 1984 to 434 in 1993. As a response, the Freedom of Access to Clinic Entrances Act was passed in May, 1994. This Act makes it a federal offense to physically obstruct the entrance of an abortion clinic or to use force, threats, or tactics intended to intimidate women seeking abortions. |
| (Graff and Amoyaw 2009) [Ghana] | Situational analysis to identify barriers to sustainable MVA supply. | Literature review; stakeholders involved with MVA policy, manufacturing, procurement, training, and provision (n=70). | Situational assessment | Meso | Additional barriers included: negative abortion stigma; high cost of equipment; lack of preferred MVA_ model availability; limited access to MVA trainings; and lack of priority when facility funds are limited. |
| (Grindlay, Lane et al. 2013) [United States] | The purpose of this study was to evaluate patients’ and providers’ experiences with telemedicine provision of medical abortion. | Women receiving telemedicine (n=20) or in-person (n=5) medical abortion services from Planned Parenthood of Heartland clinics in Iowa. | Qualitative: in-depth interviews | Micro | Some participants felt more strongly about their preferences, with several stating that they would choose telemedicine over an in-person visit if all other factors were equal. In two cases, this was because of internalized or anticipated stigma related to the abortion that made it feel easier for the women to talk to the doctor in a more removed manner. Internalized abortion stigma seemed to color some women’s perceptions of the telemedicine service. For a few women, the anonymity provided by the videoconference communication insulated them from the interaction with the physician, which they anticipated to be negative, and allowed them to maintain a degree of secrecy about the procedure even from their provider. |
| (Grossman, Ellertson et al. 2004) [Global] | Review of protocols and existing evidence on follow-up care for abortion. | Global literature on follow-up visits for abortion care. | Literature review | Micro; Meso | Transient feelings of guilt, sadness, or loss are common, but no evidence indicates that routine counseling is essential or even beneficial in coping with these feelings. More serious psychiatric illness after abortion occurs in women with a history of psychiatric illness. Risk factors for more serious postabortion psychosocial difficulties, including coercion to either have or not have the abortion, a genetic or medical indication for the abortion, lack of social supports, and women who demonstrate paralyzing ambivalence about the procedure. Even more difficult to quantify are the potential emotional costs of a postabortion follow-up visit. Women who return for a follow-up visit at an abortion clinic may be forced yet again to cross picket lines, an unfortunate feature of 80% of large facilities that provide abortion. |
| (Guttmacher, Kapadia et al. 1998) [South Africa] | Article examines the policies that have regulated accessibility of abortion and assesses their impact on reproductive health. | South African policies. | Review of policies and related evidence | Meso; Macro | Under the new act, health care workers are not mandated to perform abortions, or even to refer women to other providers. Their only obligation is to inform women of their rights under the new law. Thus, lack of cooperation by health care workers claiming conscientious objection due to moral or religious conflicts is emerging as a major obstacle for women seeking abortion services. |
| (Harries, Lince et al. 2012) [South Africa] | To better understand what doctors, nurses and hospital managers involved in second trimester abortion care thought about these services and how they could be improved | Abortion-related service providers and managers in the Western Cape Province, South Africa (n=19) | Qualitative: In-depth interviews | Meso | The contentious nature of abortion provision resulted in many providers being stigmatized in their workplace. Stigma was often heightened with second trimester abortion services compared with ﬁrst trimester services. Related to this abortion services were often located in hidden or difﬁcult to access places where providers who worked there were viewed as performing the ‘devil’s work’, as one nursing manager recounted. |
| (Haas-Wilson 1996) [Zambia] | Estimate the impact of enforced abortion restrictions on minors' demand for abortion services between 1978-1990 | Minors who sought an abortion | Regression analysis | Macro | The proxy measuring the enactment of unenforceable state restrictions, REGS, appears to increase both ABORTIONS/MINORS and ABORTIONS/BIRTHS. The proxy measuring the presence of religious opposition to abortion in the state, BELIEF, appears to decrease ABORTIONS/MINORS, but increase ABORTIONS/BIRTHS. A possible explanation of this result is that increases in BELIEF may be associated with fewer abortions obtained by minors and fewer births to minors, but BELIEF is unrelated to the number of minors in a particular state. |
| (Henshaw 1995) [United States] | To provide information on the percentage of women who travel long distances to obtain abortion services, the availability of abortion providers for second trimester services, the need to make more than one trip to the abortion facility and the amount abortion providers charge for services. In addition, it presents a measure of antiabortion harassment. | Abortion providers in the United States (n= 1,525) | Cohort | Meso | Another barrier facing many women seeking abortion services and the ability of facilities to provide services is harassment by antiabortion protesters. As in earlier rounds, this study’s 1993 survey asked providers to indicate the number of times they had experienced various types of harassment during the previous year. In all, 55% of nonhospital providers reported experiencing at least one of the 10 listed types of harassment during 1992. Harassment is strongly associated with the abortion caseload, with 86% of facilities that performed 400 or more abortions in 1992 reporting some harassment, compared with 29% of providers with a smaller caseload. The questionnaire for nonhospital providers asked for a list of the major problems that affected their ability to provide abortion services over the previous 12 months. Their responses indicate that providers see harassment and other expressions of antiabortion sentiment in the community as their most important problem with respect to abortion. Other referenced problems included picketing (8%), demonstrations and blockades (5%), vandalism and other direct action (8%) and other types or effects of harassment (8%). An additional 11% mentioned the physician shortage or other stafﬁng problems that may be indirectly related to harassment, and 2% named lack of cooperation of police or other authorities. |
| Htay 2003 [Myanmar] | Describes the process undertaken by the Department of Health (DOH) in Myanmar to address the issue of abortion complications, by integrating post-abortion care and contraceptive service delivery into existing health care services | Health-providers [n=285] and post-aboriton women [n=170]. | Cross-sectional descriptive survey | Micro; Meso | Study results found that village women tended to delay seeking care for post-abortion complications after an induced abortion for two main reasons — fear of neighbours knowing and fear that health staff in the hospital would blame them. Preliminary post-intervention research has shown that involving midwives in these ways has had positive results, with midwives having improved knowledge and confidence to counsel women post-abortion. Midwives felt that women welcomed their visits and the information they provided. The attitudes of the midwives towards women post-abortion were also much more sympathetic than prior to training. |
| (Hulme-Chambers, Temple-Smith et al. 2018) [Australia] | To understand rural women’s experiences in obtaining a medical termination of pregnancy (MToP) through a rural primary healthcare service in Victoria, Australia. | Women aged 16 years and over who attended clinic between February 2016 and 2017 for an appointment related to MToP. [n=18] | Qualitative [semi-structured interviews] | Micro; Meso | A small number of women reported feeling that their GP was obstructionist about MToP referral. As part of the MToP process, women needed to have an ultrasound to confirm intrauterine pregnancy and to obtain the mifepristone and misoprostol from the one pharmacy that the clinic has an arrangement with to stock it. Almost half of women discussed upsetting experiences when obtaining an ultrasound. Women reported feeling like the pharmacist or staff were judgmental as well. |
| (Izugbara, Egesa et al. 2015) [Kenya] | How, in the context of Kenya's current abortion law as well as severe abortion stigma in the country, do ordinary women perceive and understand abortion safety? How do lay and public health discourses of abortion safety compare?" | A convenience sample of 50 women treated for complications of unsafe abortion at six purposively-selected public facilities in Kenya. | Qualitative cross-sectional descriptive | Micro; Meso | The women we studied generally viewed induced abortion as a problematic and morally-contentious issue that was not permissible in Kenya. Generally, participants felt that they had engaged in a deviant and problematic behavior by procuring an abortion. Hospital-based providers were reportedly condemnatory and judgmental towards women seeking abortion. They reportedly gossiped about women among themselves, called them names and even publicized their abortion. Providers and facilities that act as accomplices and coconspirators with the women were considered key to abortion safety. In the current study, women's abortion safety notions underscored their anxieties, struggles and concerns as everyday people negotiating both an intensely stigmatizing behavior as well as an unsympathetic health system. |
| (Izugbara and Ukwayi 2003) [Nigeria] | To profile the characteristics and health conditions of the clientele of traditional birth homes (TBHs) in four rural communities in southeastern Nigeria | Users of TBHs and traditional birth attendants (TBAs) in rural southeastern Nigeria | Qualitative interviews | Micro; Meso | Across all age categories, the need for secrecy and privacy over condition was the most frequently mentioned reason for using TBH services. Many of the users noted that privacy was unlikely in hospitals. |
| (Jejeebhoy, Kalyanwala et al. 2010) [India] | To shed light on the experiences of unmarried young abortion-seekers aged 15–24, compare their experiences with those of their married counterparts, and explore the proximate factors leading to delays in them obtaining abortions into the second trimester. | A survey of abortion seekers and in-depth interviews with selected unmarried survey respondents [n=795 young women were surveyed: n= 549 unmarried, n=246 married]. In-depth interviews with n=26 randomly selected unmarried survey respondents. Sampled from facilities in two poorly developed neighbouring states in north India with weak health systems. | Cross sectional descriptive: survey and qualitative | Micro | Unmarried young women who reported each of the experiences underlying delayed abortion fared far worse. They were, for example, three to five times more likely than married young women to undergo second trimester abortion if they failed to recognise pregnancy by the second month, if they were  excluded from decision-making. |
| (Kishen and Stedman 2010) [Global] | Study examines evidence to suggest that the outcome of first-trimester abortions performed by suitably trained non-medical practitioners is comparable in terms of safety and efficacy to abortions performed by doctors. | Literature and data on national averages of costs to obtain an abortion. | Literature review | Meso | In South Africa, where abortion access has been addressed through trained-midwife participation, some midwives involved in providing abortion services report that they do not always receive the necessary equipment, supplies and supervision they need; that some of their colleagues, as well as members of the community, harass and intimidate them for offering this service. |
| (Margo, McCloskey et al. 2016) [United States] | To examine the impact of state policies along with other barriers when they seek abortion care | Women in South Carolina | Qualitative | Micro | Some participants anticipated stigmatizing reactions  from others, which led to self-protective secretive behavior. One woman compared such reactions to mental health care stigma, saying that she would readily explain her work absence to a colleague if abortion were viewed like any other medical procedure, but as the situation stands, it felt risky to do so. |
| (Marlow, Wamugi et al. 2014) [Kenya] | To understand the methods married women aged 24–49 and young, unmarried women aged ≤ 20 used to induce abortion, the providers they utilized and the social, economic and cultural norms that influenced women’s access to safe abortion services in Bungoma and Trans Nzoia counties in western Kenya. | Focus groups [n=10] conducted in Trans Nzoia and Bungoma county with un/married and younger/older women in rural and urban settings. | Qualitative cross-sectional descriptive | Micro; Meso | When women in both ages groups in the study were asked how a woman known or thought to have had an abortion would be treated by her community, all the focus groups discussed how women are ostracized, labelled and stigmatized as killers or murderers, are perceived to be a bad influence on others, are called prostitutes and accused of being unfaithful to their husbands or boyfriends. Younger women are perceived to be poor candidates for marriage. |
| (Mutua, Manderson et al. 2018) [Kenya] | To illustrate how the quality of PAC in healthcare facilities is impacted by law and government policy. | Patients [n=21] and providers [n=16] at 16 hospitals in three regions sampled purposively by regional area, level, and reported quality of care. | Qualitative cross-sectional | Micro; Meso; Macro | Some providers also spoke of disrespect to patients (from providers other than themselves) and argued the need for positive attitudes by health providers to encourage timely seeking of PAC services. These attitudes have also been associated with the high incidence of unsafe abortion in the country, and severe complications when women opt to self-medicate complications from unsafe terminations. In addition to age, discrimination occurred on the basis of marital status, with service providers likely to conclude that an abortion was induced if a patient was unmarried. Unmarried patients were therefore more likely to lie about their marital status, in order to gain access to the same level of care as provided to married patients. In addition to delays in seeking care occasioned by legal challenges, as discussed earlier, other forms of service unavailability and stigmatization influenced general patterns of healthcare seeking. |
| (Mutungi, Wango et al. 1999) [Kenya] | To evaluate the adolescents' behavior regarding induced abortion | Adolescent girls and boys ages 10-19 (n=1820 adolescent, 1048 school girls, 580 school boys, and 192 post abortion patients) | Cross-sectional prospective study | Micro | Many boys shy away from any responsibility and abandon their girlfriends once the girls become pregnant. |
| (Penfold, Wendot et al. 2018) [Kenya] | To explore the pathways, decision-making, experiences and preferences of women receiving safe abortion and post-abortion family planning (PAFP) at private clinics in western Kenya. | Women [n=22] who had received an abortion or post-abortion care service at one of nine clinics. | Qualitative cross-sectional | Micro | Most respondents were satisfied with the clinic and the service they received and women commonly recalled feeling relieved after completing the abortion. However, in a few cases clients recalled a negative experience at certain clinics, being made to feel “guilty”. |
| (Raifman, Anderson et al. 2018) [United States] | To explore capacity of University of California (UC) and California State University (CSU) student health centers (SHCs) to provide medication abortion (MA) and SHC staff perspectives on providing MA. | Providers and staff at student health center | Mixed methods | Meso | Interview respondents indicated that a fear of protesters and a lack of providers could be a barrier for students seeking abortion services. |
| (Robson, Kelly et al. 2009) [United Kingdom] | To determine the acceptability, efficacy and costs of medical termination of pregnancy (MTOP) compared with surgical termination of pregnancy (STOP) at less than 14 weeks’ gestation, and to understand women’s decision-making processes and experiences when accessing the termination service. | Women accepted for termination of pregnancy (TOP) with pregnancies < 14 weeks’ gestation on the day of abortion. | A partially randomised preference trial and economic evaluation with follow-up at 2 weeks and 3 months. | Micro | Once women were referred to the hospital, some had trouble locating the clinic and were reluctant to ask for directions, suggesting a degree of stigma attached to attending the TOP clinic. |
| (Sethna and Doull 2013) [Canada] | To analyse the travel women undertake to access abortion services at freestanding clinics. | Women [n=1186] seeking abortion care at 17 freestanding abortion clinics. | Cross-sectional survey and mapping | Meso | Hospital-based abortion services are essential to women living in some rural and remote communities as they may be the only point of health care access in their community. They may also provide women seeking abortions with a level of safety that can be jeopardized by protesters at freestanding abortion clinics. At the same time, women may wish to avoid hospital-based abortion services because of confidentiality issues, particularly in smaller centers. Anti-abortion hospital staff may also deliberately mislead women about the availability of local abortion services or take a judgemental approach to women seeking abortions. |
| Shah 2014 [Global] | To review the evidence on abortion laws and policies, and trends in the incidence of safe and unsafe abortion and in mortality due to unsafe abortion | Abortion seekers, countries with abortion policies | Review | Micro | Stigma is among the reasons mentioned for seeking an unsafe abortion. Given that these deaths or complications occur following a clandestine or illegal procedure, stigma and fear of punishment deter women and their families from reporting the procedure. |
| (Singh 2010) [Global] | To review the scientific evidence on the consequences of unsafe abortion, highlight gaps in the evidence base, suggest areas where future research efforts are needed, and speculate on the future situation regarding consequences and evidence over the next 5–10 years. | Women who had unsafe abortions. | Review | Micro; Meso | Stigma can be very consequential for unmarried and young women because of the strong social sanctions against sexual activity among these groups, as well as their lack of resources and inexperience in seeking healthcare. Potential consequences for unmarried young women suspected of having had an abortion include difficulty finding a partner to marry. Married women may also experience stigma because their husband and others may suspect them of infidelity; sociopsychological consequences can also be important, and may result from the attitudes of others, as well as from individuals’ own feelings of guilt and shame. |
| (Wainer 2008) [Australia] | To illuminate an important clinical question that had been inaccessible to researchers until the 1970s: What effect did an abortion have on normally rule abiding women? | Women seeking abortion services | Interviews | Micro | In the 1970s, women who were sexually active and avoided visible pregnancy, either by contraception or abortion, were better able to manage their identity as a good person than a single woman with a visible pregnancy, and so abortion can become a tool of identity management. The study participants produced four descriptions of abortion as an event based on what they thought community beliefs would be. These were that abortion was morally wrong, that it was dangerous, that is was illegal, and that it was necessary/acceptable. |
| (Whittaker 2002) [Thailand] | To examine the effects of the current laws through the experiences of women who have undergone illegal abortions. | Women of reproductive age | Qualitative | Micro | Among the study participants interviewed, abortion is considered to be a life-destroying act that constitutes a serious Buddhist sin/demerit. Many women cited fear of bap (Buddhist demerit) as the reason why they chose to continue with an unplanned pregnancy. |

Agadjanian, V. (2002). "Is "Abortion Culture" Fading in the Former Soviet Union? Views about Abortion and Contraception in Kazakhstan." Studies in Family Planning **33**(3): 237-248.

Aiken, A., R. Gomperts and J. Trussell (2017). "Experiences and characteristics of women seeking and completing at-home medical termination of pregnancy through online telemedicine in Ireland and Northern Ireland: a population-based analysis." Bjog **124**(8): 1208-1215.

Aiken, A. R. A., K. A. Guthrie, M. Schellekens, J. Trussell and R. Gomperts (2018). "Barriers to accessing abortion services and perspectives on using mifepristone and misoprostol at home in Great Britain." Contraception **97**(2): 177-183.

Aiken, A. R. A., D. M. Johnson, K. Broussard and E. Padron (2018). "Experiences of women in Ireland who accessed abortion by travelling abroad or by using abortion medication at home: a qualitative study." BMJ Sex Reprod Health.

Altshuler, A. L., A. Ojanen-Goldsmith, P. D. Blumenthal and L. R. Freedman (2017). "A good abortion experience: A qualitative exploration of women's needs and preferences in clinical care." Social Science & Medicine **191**: 109-116.

Aniteye, P. and S. Mayhew (2011). "Attitudes and Experiences of Women Admitted to Hospital with Abortion Complications in Ghana." African Journal of Reproductive Health / La Revue Africaine de la Santé Reproductive **15**(1): 47-55.

Appiah-Agyekum, N. N. (2018). "Medical abortions among university students in Ghana: implications for reproductive health education and management." International Journal of Womens Health **10**: 515-522.

Appiah-Agyekum, N. N., C. Sorkpor and S. Ofori-Mensah (2015). "Determinants of abortion decisions among Ghanaian university students." Int J Adolesc Med Health **27**(1): 79-84.

Baird, B. (2015). "Medical abortion in Australia: a short history." Reproductive Health Matters **23**(46): 169-176.

Banerjee, S. K., K. L. Andersen and J. Warvadekar (2012). "Pathways and consequences of unsafe abortion: a comparison among women with complications after induced and spontaneous abortions in Madhya Pradesh, India." International Journal of Gynecology & Obstetrics **118**: S113-120.

Brack, C. E., R. W. Rochat and O. A. Bernal (2017). "It's a Race Against the Clock: A Qualitative Analysis of Barriers to Legal Abortion in Bogot, Colombia." International Perspectives on Sexual and Reproductive Health **43**(4): 173-182.

Casas-Becerra, L. (1997). "Women prosecuted and imprisoned for abortion in Chile." Reproductive Health Matters **5**(9): 29-36.

Casas, L. and L. Vivaldi (2014). "Abortion in Chile: the practice under a restrictive regime." Reproductive Health Matters **22**(44): 70-81.

Chevrette, M. and H. A. Abenhaim (2015). "Do State-Based Policies Have an Impact on Teen Birth Rates and Teen Abortion Rates in the United States?" Journal of Pediatric and Adolescent Gynecology **28**(5): 354-361.

Chunuan, S., S. Kosunvanna, W. Sripotchanart, J. Lawantra, J. Lawantrakul, U. Pattrapakdikul and J. Somporn (2012). "Characteristics of Abortions in Southern Thailand." Pacific Rim International Journal of Nursing Research **16**(2): 97-112.

Contreras, X., M. G. van Dijk, T. Sanchez and P. S. Smith (2011). "Experiences and Opinions of Health-Care Professionals Regarding Legal Abortion in Mexico City: A Qualitative Study." Studies in Family Planning **42**(3): 183-190.

Cook, S., B. de Kok and M. L. Odland (2017). "'It's a very complicated issue here': understanding the limited and declining use of manual vacuum aspiration for postabortion care in Malawi: a qualitative study." Health Policy & Planning **32**(3): 305-313.

David, H. P. and A. Baban (1996). "Women's health and reproductive rights: Romanian experience." Patient Education & Counseling **28**(3): 235-245.

de Bruyn, M. (2003). "Safe Abortion for HIV-Positive Women with Unwanted Pregnancy: A Reproductive Right." Reproductive Health Matters **11**(22): 152-161.

Diniz, S. G., A. F. P. L. d'Oliveira and S. Lansky (2012). "Equity and women's health services for contraception, abortion and childbirth in Brazil." Reproductive Health Matters **20**(40): 94-101.

Dobie, S. A., L. Gober and R. A. Rosenblatt (1998). "Family planning service provision in rural areas: a survey in Washington State." Fam Plann Perspect **30**(3): 139-142, 147.

Doran, F. and S. Nancarrow (2015). "Barriers and facilitators of access to first-trimester abortion services for women in the developed world: a systematic review." Journal of Family Planning & Reproductive Health Care **41**(3): 170-180.

Doran, F. M. and J. Hornibrook (2016). "Barriers around access to abortion experienced by rural women in New South Wales, Australia." Rural & Remote Health **16**(1): 3538-3549.

Esia-Donkon, K., E. K. M. Darteh, H. Blemano and H. Asare (2015). "Who Cares? Pre and Post Abortion Experiences among Young Females in Cape Coast Metropolis, Ghana." African Journal of Reproductive Health / La Revue Africaine de la Santé Reproductive **19**(2): 43-51.

Finer, L. B., L. F. Frohwirth, L. A. Dauphinee, S. Singh and A. M. Moore (2005). "Reasons U.S. Women Have Abortions: Quantitative and Qualitative Perspectives." Perspectives on Sexual and Reproductive Health **37**(3): 110-118.

Foster, A. M., K. J. LaRoche, J. El-Haddad, L. DeGroot and I. M. El-Mowafi (2017). ""If I ever did have a daughter, I wouldn't raise her in New Brunswick:" exploring women's experiences obtaining abortion care before and after policy reform." Contraception **95**(5): 477-484.

Gerdts, C., S. Raifman, K. Daskilewicz, M. Momberg, S. Roberts and J. Harries (2017). "Women's experiences seeking informal sector abortion services in Cape Town, South Africa: a descriptive study." BMC Women's Health **17**: 1-10.

Glenton, C., A. M. Sorhaindo, B. Ganatra and S. Lewin (2017). "Implementation considerations when expanding health worker roles to include safe abortion care: a five-country case study synthesis." BMC Public Health **17**: 1-13.

Gober, P. (1997). "The role of access in explaining state abortion rates." Social Science & Medicine **44**(7): 1003-1016.

Graff, M. and D. A. Amoyaw (2009). "Barriers to Sustainable MVA Supply in Ghana: Challenges for the Low-Volume, Low-Income Providers." African Journal of Reproductive Health / La Revue Africaine de la Santé Reproductive **13**(4): 73-80.

Grindlay, K., K. Lane and D. Grossman (2013). "Women's and Providers' Experiences with Medical Abortion Provided Through Telemedicine: A Qualitative Study." Women's Health Issues **23**(2): e117-e122.

Grossman, D., C. Ellertson, D. A. Grimes and D. Walker (2004). "Routine follow-up visits after first-trimester induced abortion." Obstet Gynecol **103**(4): 738-745.

Guttmacher, S., F. Kapadia, N. Jim Te Water and H. de Pinho (1998). "Abortion Reform in South Africa: A Case Study of the 1996 Choice on Termination of Pregnancy Act." International Family Planning Perspectives **24**(4): 191-194.

Haas-Wilson, D. (1996). "The Impact of State Abortion Restrictions on Minors' Demand for Abortions." Journal of Human Resources **31**(1): 140-158.

Harries, J., N. Lince, D. Constant, A. Hargey and D. Grossman (2012). "The challenges of offering public second trimester abortion services in South Africa: health care providers' perspectives." Journal of Biosocial Science **44**(2): 197-208.

Henshaw, S. K. (1995). "Factors hindering access to abortion services." Family Planning Perspectives **27**(2): 54-87.

Hulme-Chambers, A., M. Temple-Smith, A. Davidson, L. Coelli, C. Orr and J. E. Tomnay (2018). "Australian women’s experiences of a rural medical termination of pregnancy service: A qualitative study." Sexual & Reproductive Healthcare **15**: 23-27.

Izugbara, C. O., C. Egesa and R. Okelo (2015). "‘High profile health facilities can add to your trouble’: Women, stigma and un/safe abortion in Kenya." Social Science & Medicine **141**: 9-18.

Izugbara, C. O. and J. K. Ukwayi (2003). "The clientele of traditional birth homes in rural southeastern Nigeria." Health Care for Women International **24**(3): 177-192.

Jejeebhoy, S. J., S. Kalyanwala, A. J. F. Zavier, R. Kumar and N. Jha (2010). "Experience seeking abortion among unmarried young women in Bihar and Jharkhand, India: delays and disadvantages." Reproductive Health Matters **18**(35): 163-174.

Kishen, M. and Y. Stedman (2010). "The role of Advanced Nurse Practitioners in the availability of abortion services." Best Practice & Research Clinical Obstetrics & Gynaecology **24**(5): 569-578.

Margo, J., L. McCloskey, G. Gupte, M. Zurek, S. Bhakta and E. Feinberg (2016). "Women's Pathways to Abortion Care in South Carolina: A Qualitative Study of Obstacles and Supports." Perspectives on Sexual & Reproductive Health **48**(4): 199-207.

Marlow, H. M., S. Wamugi, E. Yegon, T. Fetters, L. Wanaswa and S. Msipa-Ndebele (2014). "Women’s perceptions about abortion in their communities: perspectives from western Kenya." Reproductive Health Matters **22**(43): 149-158.

Mutua, M. M., L. Manderson, E. Musenge and T. N. O. Achia (2018). "Policy, law and post-abortion care services in Kenya." Plos One **13**(9): 18.

Mutungi, A. K., E. O. Wango, K. O. Rogo, V. N. Kimani and J. G. Karanja (1999). "Abortion: Behaviour of adolescents in two districts in Kenya." East African Medical Journal **76**(10): 541-546.

Penfold, S., S. Wendot, I. Nafula and K. Footman (2018). "A qualitative study of safe abortion and post-abortion family planning service experiences of women attending private facilities in Kenya." Reproductive Health **15**(1): N.PAG-N.PAG.

Raifman, S., P. Anderson, S. Kaller, D. Tober and D. Grossman (2018). "Evaluating the capacity of California's publicly funded universities to provide medication abortion." Contraception **98**(4): 306-311.

Robson, S., T. Kelly, D. Howel, M. Deverill, J. Hewison, M. Lie, E. Stamp, N. Armstrong and C. May (2009). "Randomised preference trial of medical versus surgical termination of pregnancy less than 14 weeks' gestation (TOPS)." Health Technology Assessment **13**(37): 1-148.

Sethna, C. and M. Doull (2013). "Spatial disparities and travel to freestanding abortion clinics in Canada." Women's Studies International Forum **38**: 52-62.

Singh, S. (2010). "Global consequences of unsafe abortion." Womens Health (Lond) **6**(6): 849-860.

Wainer, J. (2008). "Abortion and the struggle to be good in the 1970s." Australian & New Zealand Journal of Psychiatry **42**(1): 30-37.

Whittaker, A. (2002). "Reproducing inequalities: abortion policy and practice in Thailand." Women & Health **35**(4): 101-119.

1. [↑](#footnote-ref-1)
