# Supplementary material for: The economics of abortion and its links with stigma: A secondary analysis from a scoping review on the economics of abortion
Source: PLoS One. 2021 Feb 18;16(2):e0246238. doi: 10.1371/journal.pone.0246238 (PMC7891754; doi:10.1371/journal.pone.0246238)
Supplement: S4 Appendix — (DOCX) [file pone.0246238.s004.docx]

**S4 Appendix.** **Summary of included studies reporting abortion-related stigma and economic costs at the microeconomic level (n=15)**

| **Author, year [country]** | **Aim/objective(s)** | **Population** | **Study type** | **Summary of main findings** |
| --- | --- | --- | --- | --- |
| (Acosta de Hart, Umaña et al. 2002) [Colombia] | To descriptively review Orientame’s service over 25 years | People living in Bogota that are seeking care | Narrative description (historical) | The study found that women with low incomes have nearly no access to qualified service providers in general. This access is further limited for stigmatized conditions like unwanted pregnancy or incomplete abortions. |
| (Ahmed, Islam et al. 1999) [Bangladesh] | To find out where women go for induced abortion in rural Bangladesh today, their contraceptive practice prior to and after getting pregnant, their reasons for choosing abortion, who makes the decision for abortion, what complications they develop, and where they go for treatment for these complications | All women seeking abortion-related care at six health facilities (two rural hospitals and four static clinics) in two rural sub-districts of Bangladesh in 1996-1997 (n=143) | Semi-structured questionnaire with interviews | One study participant claimed that the doctors and staff at the facility did not give her adequate care because she was poor. |
| (Aiken, Gomperts et al. 2017)[Ireland] | To examine the characteristics and experiences of women in Ireland and Northern Ireland seeking at-home medical termination of pregnancy (TOP) using online telemedicine | Women who requested at-home medical TOP through online telemedicine initiative Women on Web from 2010-2015 (n=5,650)  Women who completed TOP in 2010-2012 (n=1,023) | Logistic regression and content analysis of women’s evaluations | The association the authors observed between having few financial resources and reporting a lack of support could be explained by a higher likelihood of experiencing stigma in communities with lower socio-economic status or by a reverse association, where lack of ability to confide in family and friends is associated with having fewer financial resources due to the inability to borrow money for the donation. |
| (Aiken, Guthrie et al. 2018) [United Kingdom] | To examine reasons for seeking abortion services outside the formal healthcare system in Great Britain, where abortion is legally available | Women resident in England, Scotland, and Wales who requested at-home medication abortion through online telemedicine initiative Women on Web in 2016-2017 (n=519) | Mixed methods | Key categories of circumstances and reasons for accessing abortion services outside the formal healthcare setting in Great Britain (n=209): perceived or experienced stigma (12.9%). Study participants expressed feelings of shame and embarrassment to return to clinics where they have received an abortion in the past, and/or they do not want to go to a facility in a small area as there is a lack of confidentiality. |
| (Baum, White et al. 2016) [United States] | To explore qualitatively the experiences of women who were most affected by restrict abortion laws in Texas: those who had to travel farther to reach a facility and those desiring medication abortion | Women recruited from ten abortion clinics across Texas. The purposive sample included women who obtained or strongly preferred medication abortion or traveled ≥50 miles one way to the clinic. (n=20) | Qualitative, in-depth interviews | Beyond financial costs, having to travel longer distances for care imposes social costs for women. Women’s social networks were essential for helping them minimize the out-of-pocket expenses associated with their clinic visits, including borrowing a car or getting a ride and assistance with childcare. However, women who expressed fear of stigma by friends and family or ways that drawing on these networks might compromise their desire for confidentiality. |
| (Baxerres, Boko et al. 2018) [Benin, Burkina Faso] | To document the means women use to obtain abortions in the capital cities of Benin and Burkina Faso, and to learn whether or not use of misoprostol has become an alternative to other methods of abortion, and the implications for future practice. | 21 women in Cotonou and 13 women in Ouagadougou, including 5 secondary school students. | Qualitative in-depth interviews | Decision-making was often influenced by the need for secrecy, which meant that pregnancy people would travel further away from their homes to seek clinics or providers that they could consult. |
| (Bennett 2001) [Indonesia] | To examine the experience of premarital pregnancy and induced abortion among young, single women in Lombok, Eastern Indonesia | Single women and their families, and health care providers in Mataram, the capital city of Lombok, between August 1996 and February 1998 | Ethnography | This study reports that abortion-based stigma often results in poor quality of care and increased risk of morbidity for unmarried women who have unequal access to abortion services, relative to married women. The decision of a single woman to continue a premarital pregnancy is also constrained by lack of social autonomy and the absence of acceptable alternatives. |
| (Bessett, Gorski et al. 2011) [United States] | To learn about women’s experiences applying for subsidized insurance and to identify barriers to obtaining insurance or its use for abortion services | English-speaking women who met the eligibility requirements for subsidized insurance programs in Massachusetts: Women whose household income is less than 300% FPL, who are uninsured, who are U.S. citizens or have been permanent residents for more than 5 years, and who  are Massachusetts residents (n=39) | Systematic, qualitative interviews with women | A few study participants reported that concerns about abortion stigma caused them to avoid mentioning their pregnancy or their intention to obtain an abortion to state employees and insurance advocates, compounding their difficulties in navigating the application process |
| (Doran and Hornibrook 2014) [Australia] | Identify factors that New South Wales (NSW) rural women experience in relation to their ability to access an abortion service and follow-up care. | Rural clinics in NSW (n=7) and women who sought abortion services at those clinics (n=13). | Qualitative: Surveys and interviews | Reducing stigma and negative attitudes was mentioned by all study participants to improve access to appropriate care, service delivery and community understanding. One participant needed to visit five rural GPs before she received a referral and described the process as ‘demoralising’ and significantly delayed the procedure. Another participant found it challenging as she needed overnight childcare. One participant commented on ‘moralistic ideology and judgement’ around abortion and the propaganda about RU486, which she thought limited availability. Others had to travel 4 hours for this procedure when a curette could be offered in the local hospital. |
| (Ganatra and Hirve 2002) [India] | To explore adolescent women's access to abortion services, decision-making on abortion, determinants of provider choice and extent of morbidity experienced. | Women (primarily those currently married) who had undergone an induced abortion in the study area in an 18-month reference period during 1996–1998 | Questionnaires and a qualitative in-depth time-line of sequence of events | Private practitioners provided young women the confidentiality they needed and were a reasonable distance away, but the cost of their services was high and so they were accessed only by those girls with financial support from their parents or brothers. One young girl, who was asked to pay Rs. 1200 (US$25) for a first trimester abortion, was told: ‘‘Yes, I can do it. You can go home in two hours. No one will come to know. But for illegal cases like yours, I am taking a big risk. I have to charge accordingly.’’ (private provider, male)" |
| (Hung 2010) [Hong Kong] | To describe the experience of teenage women in Hong Kong from deprived backgrounds who seek an abortion and the extent to which their access to legal services is constrained by age, income and class. | Young women [n=29] from deprived backgrounds in Hong Kong who had had an abortion, and key informants [n=4] | Qualitative cross-sectional descriptive | Five of the respondents, either out of their own savings or with the financial support of family members, were able to afford the costs of private medical services, including visiting private doctors and having the abortion in a private hospital which is of better quality, less stigmatized and can better ensure privacy than using public services. |
| (Potdar, Fetters et al. 2008) [Cambodia] | To describe the loss of productive time and income related to abortion care and care-seeking. | 160 women seeking elective abortions or care for complications of abortion from a purposefully selected group of public and private clinics and hospitals. | Cross-sectional survey | The stigma that surrounds abortion prevents many women from making informed and educated choices about an unwanted pregnancy. The results of this study indicate that the amount of time and money spent accessing information and appropriate abortion care is highly variable, even in Cambodia, where abortion is technically legal and theoretically safe. Women’s inability to make informed decisions because of a lack of reliable information affects not only her health but also the direct and indirect costs of her care. |
| (Raifman, Anderson et al. 2018) | To explore capacity of University of California (UC) and California State University (CSU) student health centers (SHCs) to provide medication abortion (MA) and SHC staff perspectives on providing MA. | Providers and staff at student health center | Mixed methods | Survey responses indicated that cost, fear of protesters, lack of providers, lack of family support are all barriers to students seeking abortion services. |
| (Singh 2010) [Global] | This article will review the scientific evidence on the consequences of unsafe abortion, highlight gaps in the evidence base, suggest areas where future research efforts are needed, and speculate on the future situation regarding consequences and evidence over the next 5–10 years. | Women who obtained unsafe abortions globally. | Literature review | Studies in Uganda and Zimbabwe show similar findings regarding the attitudes of men towards women who have abortions: most men perceive that women who have had or are having abortions are doing so because they are pregnant by a man other than their husband, and state that they would not provide support (financial or otherwise) to a woman in such a situation to help her obtain the abortion or postabortion care. |
| (Wu, Maru et al. 2017) [Nepal] | To review abortion care in Nepal 15 years after it was legalized. | n/a | Review | Fear of stigma also prevents some women from seeking abortion services. According to one study focused on young women, many such women do not seek abortion for an unintended pregnancy due to several factors, including partner and family influences as well as limited socioeconomic resources. |

Acosta de Hart, H., C. Umaña and C. Villarreal (2002). "Oriéntame: Preventing and Solving Problems Related to Unwanted Pregnancy for 25 Years in Colombia." Reproductive Health Matters **10**(19): 138-142.

Ahmed, S., A. Islam, P. A. Khanum and K. Barkat e (1999). "Induced abortion: What's happening in rural Bangladesh." Reproductive Health Matters **7**(14): 19-29.

Aiken, A., R. Gomperts and J. Trussell (2017). "Experiences and characteristics of women seeking and completing at-home medical termination of pregnancy through online telemedicine in Ireland and Northern Ireland: a population-based analysis." Bjog **124**(8): 1208-1215.

Aiken, A. R. A., K. A. Guthrie, M. Schellekens, J. Trussell and R. Gomperts (2018). "Barriers to accessing abortion services and perspectives on using mifepristone and misoprostol at home in Great Britain." Contraception **97**(2): 177-183.

Baum, S. E., K. White, K. Hopkins, J. E. Potter and D. Grossman (2016). "Women's Experience Obtaining Abortion Care in Texas after Implementation of Restrictive Abortion Laws: A Qualitative Study." PLoS One **11**(10): e0165048.

Baxerres, C., I. Boko, A. Konkobo, F. Ouattara and A. Guillaume (2018). "Abortion in two francophone African countries: a study of whether women have begun to use misoprostol in Benin and Burkina Faso." Contraception **97**(2): 130-136.

Bennett, L. R. (2001). "Single women's experiences of premarital pregnancy and induced abortion in Lombok, Eastern Indonesia." Reproductive Health Matters **9**(17): 37-43.

Bessett, D., K. Gorski, D. Jinadasa, M. Ostrow and M. J. Peterson (2011). "Out of Time and Out of Pocket: Experiences of Women Seeking State-Subsidized Insurance for Abortion Care in Massachusetts." Women's Health Issues **21**(3, Supplement): S21-S25.

Doran, F. and J. Hornibrook (2014). "Rural New South Wales women's access to abortion services: highlights from an exploratory qualitative study." Aust J Rural Health **22**(3): 121-126.

Ganatra, B. and S. Hirve (2002). "Induced Abortions Among Adolescent Women in Rural Maharashtra, India." Reproductive Health Matters **10**(19): 76-85.

Hung, S. L. (2010). "Access to safe and legal abortion for teenage women from deprived backgrounds in Hong Kong." Reproductive Health Matters **18**(36): 102-110.

Potdar, R., T. Fetters and L. Phirun (2008). "Initial loss of productive days and income among women seeking induced abortion in Cambodia." Journal of Midwifery & Women's Health **53**(2): 123-129.

Raifman, S., P. Anderson, S. Kaller, D. Tober and D. Grossman (2018). "Evaluating the capacity of California's publicly funded universities to provide medication abortion." Contraception **98**(4): 306-311.

Singh, S. (2010). "Global consequences of unsafe abortion." Womens Health (Lond) **6**(6): 849-860.

Wu, W.-J., S. Maru, K. Regmi and I. Basnett (2017). "Abortion Care in Nepal, 15 Years after Legalization

Gaps in Access, Equity, and Quality." Health and Human Rights **19**(1): 221-230.
