# Supplementary material for: The economics of abortion and its links with stigma: A secondary analysis from a scoping review on the economics of abortion
Source: PLoS One. 2021 Feb 18;16(2):e0246238. doi: 10.1371/journal.pone.0246238 (PMC7891754; doi:10.1371/journal.pone.0246238)
Supplement: S5 Appendix — (DOCX) [file pone.0246238.s005.docx]

**S5 Appendix.** **Summary of included studies reporting abortion-related stigma and economic impact at the microeconomic level (n=4)**

| **Author, year [country]** | **Aim/objective(s)** | **Population** | **Study type** | **Summary of main findings** |
| --- | --- | --- | --- | --- |
| (Aiken, Johnson et al. 2018) [Ireland] | (1) to examine the factors affecting whether women in Ireland choose to access abortion by travelling or by using online telemedicine; and (2) to explore their experiences in accessing care through each pathway | Women (n=38) identified through three organisations: Women on Web, Abortion Support Network, For Reproductive Rights Against Oppression, Sexism and Austerity.  Criteria: aged over 18, had an abortion within 8 years of study, lived in Ireland at time of abortion, had travelled or used telemedicine to access abortion care. | Qualitative in-depth interviews | Fears and judgement led pregnant people in Ireland to travel for their abortions: Study participants reported that their fears in confiding with their local doctor would negatively impact their relationship, and were therefore motivated to travel to receive an abortion. |
| (Bennett 2001) [Indonesia] | To examine the experience of premarital pregnancy and induced abortion among young, single women in Lombok, Eastern Indonesia | Single women and their families, and health care providers in Mataram, the capital city of Lombok, between August 1996 and February 1998 | Ethnography | Participants in this study felt that while abortion was a sin, it was also acceptable in certain circumstances. In the focus group discussions, they tended to focus on whether marriage was possible and the wishes of the man involved. Many felt that abortion was preferable to continuing a pregnancy if he refused to take responsibility or rejected marriage as a solution. Study participants often argued that causing personal and family shame, having a child out of wedlock and raising a fatherless child were greater sins than abortion. |
| (Brack, Rochat et al. 2017) [Colombia] | To identify the key barriers to legal abortion, and to explore the ways they may work separately and together to delay the receipt of high quality, legal abortion care | Women who had obtained a legal abortion in Bogotá, Colombia (n=17). Women were eligible for inclusion if they were aged 18 or older, had obtained an abortion in the past 12 months and exhibited verbal proficiency in Spanish | Qualitative: In-depth interviews with women | Three study participants described the stigma attached to having a child, in contrast to the stigma of having an abortion, given their current educational endeavors, and romantic and financial situations. They noted that in Colombian society, people pass judgment on single mothers and think poorly of them for having to drop out of school, for having a child out of wedlock and for being unable to afford the child. These participants stated that their families would also judge them for these reasons. |
| (Moore, Dennis et al. 2018) [Zambia] | To compare the financial costs for women when they have an induced abortion at a facility, with costs for an induced abortion outside a facility, followed by care for abortion-related complications | Women seeking care at two public hospitals and two private clinics, one each in Lusaka and in Kafue districts in Zambia. The data were collected from women (n = 38) obtaining a legal termination of pregnancy (TOP), or care for unsafe abortions (CUA). | Mixed methods: household wealth data at one point in time (T1) and longitudinal qualitative data at two points in time (T1 and T2, three-four months later), in Lusaka and Kafue districts, between 2014 and 2015. | In some cases, other costs were opportunity costs which were incurred as the news of the abortion spread and stigma was enacted on the woman in her social or professional life. One woman described being “chased” from her job because of her abortion, and had to find a new job elsewhere. |

Aiken, A. R. A., D. M. Johnson, K. Broussard and E. Padron (2018). "Experiences of women in Ireland who accessed abortion by travelling abroad or by using abortion medication at home: a qualitative study." BMJ Sex Reprod Health.

Bennett, L. R. (2001). "Single women's experiences of premarital pregnancy and induced abortion in Lombok, Eastern Indonesia." Reproductive Health Matters **9**(17): 37-43.

Brack, C. E., R. W. Rochat and O. A. Bernal (2017). "It's a Race Against the Clock: A Qualitative Analysis of Barriers to Legal Abortion in Bogot, Colombia." International Perspectives on Sexual and Reproductive Health **43**(4): 173-182.

Moore, A. M., M. Dennis, R. Anderson, A. Bankole, A. Abelson, G. Greco and B. Vwalika (2018). "Comparing women's financial costs of induced abortion at a facility vs. seeking treatment for complications from unsafe abortion in Zambia." Reproductive Health Matters **26**(52): 138-150.
