# Supplementary material for: The economics of abortion and its links with stigma: A secondary analysis from a scoping review on the economics of abortion
Source: PLoS One. 2021 Feb 18;16(2):e0246238. doi: 10.1371/journal.pone.0246238 (PMC7891754; doi:10.1371/journal.pone.0246238)
Supplement: S6 Appendix — (DOCX) [file pone.0246238.s006.docx]

**S6 Appendix.** **Summary of included studies reporting abortion-related stigma and economic benefits at the microeconomic level (n=1)**

| **Author, year [country]** | **Aim/objective(s)** | **Population** | **Study type** | **Summary of main findings** |
| --- | --- | --- | --- | --- |
| (Svanemyr and Sundby 2007) [Cote d'Ivoire] | To analyse how illegally induced abortion is understood in terms of social processes. | Women seeking care for abortion complications and key informants | Qualitative cross-sectional | Muslim women are seeking to give priority to education and financial independence so they prefer abortion even though this is strongly in opposition to their values and norms. |

Svanemyr, J. and J. Sundby (2007). "The Social Context of Induced Abortions among Young Couples in Côte d'Ivoire." African Journal of Reproductive Health / La Revue Africaine de la Santé Reproductive **11**(2): 13-23.
