# Supplementary material for: The economics of abortion and its links with stigma: A secondary analysis from a scoping review on the economics of abortion
Source: PLoS One. 2021 Feb 18;16(2):e0246238. doi: 10.1371/journal.pone.0246238 (PMC7891754; doi:10.1371/journal.pone.0246238)
Supplement: S7 Appendix — (DOCX) [file pone.0246238.s007.docx]

**S7 Appendix.** **Summary of included studies reporting abortion-related stigma and economic costs at the mesoeconomic level (n=10)**

| **Author, year [country]** | **Aim/objective(s)** | **Population** | **Study type** | **Summary of main findings** |
| --- | --- | --- | --- | --- |
| (Bennett 2001) [Indonesia] | To examine the experience of premarital pregnancy and induced abortion among young, single women in Lombok, Eastern Indonesia | Single women and their families, and health care providers in Mataram, the capital city of Lombok, between August 1996 and February 1998 | Ethnography | This study has found that the inflated fees from providers, unregulated because abortion procedures are clandestine, is a significant barrier to services. To ensure confidentiality, unmarried women will present to private medical providers for treatment. Costs of service can range from 60,000 – 300,000 rupiah, with purchase of drugs as an additional, separate cost. The high price point for confidentiality is often beyond the independent financial resources of most single women. |
| (Brack, Rochat et al. 2017) [Colombia] | To identify the key barriers to legal abortion, and to explore the ways they may work separately and together to delay the receipt of high quality, legal abortion care | Women who had obtained a legal abortion in Bogotá, Colombia in the last 12 months; were aged 18 or older; and exhibited verbal proficiency in Spanish (n=17) | In-depth interviews | In general, insurance companies acted as a barrier to timely access to legal, safe abortion care, even though they are legally obligated to authorize the procedure. Religious sentiments appeared to underlie the behavior of company representatives. |
| (Chełstowska 2011) [Poland] | To describe the economic consequences of the stigmatisation and illegality of abortion and its almost complete removal from public health services in Poland since the late 1980s. | Polish women | Review | In the private sector, illegal abortion must be cautiously arranged and paid for out of pocket. The more abortion is stigmatised in the public sphere, the more women depend on the private sector for solutions. In Poland it is common for doctors to deny abortion care to women whose health or life is in danger, because doctors count on them to “cope” with health problems privately. A pregnant woman cannot be sure whether a doctor who issues an opinion about her pregnancy is guided by what is good for her, or by their own apprehension, prejudice or interest. Some of the same doctors will refuse to perform abortions in public hospitals just to take that risk when a woman comes to their private practice. Private practice of abortion services is a vast, untaxed source of income. That is why the medical profession is not interested in changing the abortion law. |
| (Coast and Murray 2016) [Zambia] | To analyse the details of pathways to care, barriers and delays, and the role of others in influencing these pathways. | Females aged 15-43 years seeking either safe abortion or PAC at University Teachig Hospital, Lusaka [n=112] | Qualitative cross-sectional | Stigma and the false perception that abortion is illegal contribute to steering women towards clandestine methods, and reinforce women's silence and isolation. Abortion stigma gives providers leverage to extort unofficial and illegal fees, knowing that women are desperate for the service and unlikely to expose these financial demands for fear of revealing an abortion. There was also a difficult-to-quantify undercurrent of unofficial private practice that exploited the stigma surrounding abortion and women's lack of clarity on their entitlement to services. Women can find it difficult to find out how much abortion care will cost; even legal service providers do not advertise their abortion service charges openly, unlike other sexual health services. When unofficial payments are paid, secrecy means that there can be substantial gaps between expected and actual costs. |
| (Díaz-Olavarrieta, Cravioto et al. 2012) [Mexico] |  | Key informants: clinic health providers [n=10], hospital health providers [n=9] |  | A study reported that 38% of providers were opposed to provide abortion services for free because they needed to compensate the high costs of the program and create a dissuasive element to avoid abuses. In this study, the mechanism suggested by the participants was to implement a payments system in the rest of the public services that provide this service. |
| (Ganatra and Hirve 2002) [India] | To explore adolescent women's access to abortion services, decision-making on abortion, determinants of provider choice and extent of morbidity experienced. | Women (primarily those currently married) who had undergone an induced abortion in the study area in an 18-month reference period during 1996–1998 | Questionnaires and a qualitative in-depth time-line of sequence of events | Providers routinely referred to unmarried adolescents as ‘‘illegal’’ cases because their pregnancies did not occur within the context of marriage. While we did not ask them about the costs of services for unmarried clients, a few providers did mention that unmarried young women are charged three to five times the normal rate. Some 40% selectively refused services to unmarried and separated young women even though they offered them to other women. |
| (Gober 1997) [United States] | Investigate the role of access in explaining the variation in state abortion rates | U.S. state policies and abortion rates | Regression analysis | Each year a growing number of clinicians are unwilling to perform abortions, citing as reasons poor pay, low prestige, harassment and intimidation, suboptimal working conditions, the tedium of performing largely repetitive operations, and emotional stress surrounding unwanted pregnancies and families in crisis.  The public controversy surrounding abortion renders it unlike other medical procedures. Clinic personnel are stalked and taunted by antiabortion demonstrators. Doctors who perform abortions risk their physical safety, the loss of hospital privileges, and vigorous scrutiny by state licensing boards. The result is to isolate the practice of abortion from other aspects of reproductive health and to recast the public perception of abortion from a routine, safe, and inexpensive medical procedure to a burdensome, socially stigmatized act. |
| (Henshaw 1995) [United States] | To provide information on the percentage of women who travel long distances to obtain abortion services, the availability of abortion providers for second trimester services, the need to make more than one trip to the abortion facility and the amount abortion providers charge for services. In addition, it presents a measure of antiabortion harassment. | Abortion providers in the United States (n= 1,525) | Cohort | Another type of problem faced by facilities, named by 6% or provider respondents, concerned regulation and legislation at the national and state level, especially the denial of Medicaid payment for abortion. 6% identiﬁed business problems, including reduced demand, competition and rising costs, and 1% cited insurance problems. |
| (Marlow, Wamugi et al. 2014) [Kenya] | To understand the methods married women aged 24–49 and young, unmarried women aged ≤ 20 used to induce abortion, the providers they utilized and the social, economic and cultural norms that influenced women’s access to safe abortion services in Bungoma and Trans Nzoia counties in western Kenya. | Focus groups [n=10] conducted in Trans Nzoia and Bungoma county with un/married and younger/older women in rural and urban settings. | Qualitative cross-sectional descriptive | The secrecy and perceived illegality of abortion  as a stigmatized, illicit, back-door activity, keeps it out of the public purview. One study participant said that some providers can help but you cannot openly ask them for an abortion; you have to ask them to do it outside of the hospital. Married women from Bungoma and Trans Nzoia discussed arranging an abortion as a negotiable financial transaction that depends on the demand on a given day and whether the woman looks like she has money. |
| (Upadhyay, Johns et al. 2017) [United States] | To examine the association between distance travelled for an abortion and site of PAC among low-income women | Women seeking abortion services who were beneficiaries of the California Medicaid program | Retrospective observational cohort study | This study found that post-abortion visits to the emergency room (ED) as opposed to other facility types or visits may be driven by stigma, worry, or distrust of providers. |

Bennett, L. R. (2001). "Single women's experiences of premarital pregnancy and induced abortion in Lombok, Eastern Indonesia." Reproductive Health Matters **9**(17): 37-43.

Brack, C. E., R. W. Rochat and O. A. Bernal (2017). "It's a Race Against the Clock: A Qualitative Analysis of Barriers to Legal Abortion in Bogot, Colombia." International Perspectives on Sexual and Reproductive Health **43**(4): 173-182.

Chełstowska, A. (2011). "Stigmatisation and commercialisation of abortion services in Poland: turning sin into gold." Reproductive Health Matters **19**(37): 98-106.

Coast, E. and S. F. Murray (2016). "“These things are dangerous”: Understanding induced abortion trajectories in urban Zambia." Social Science & Medicine **153**: 201-209.

Díaz-Olavarrieta, C., V. M. Cravioto, A. Villalobos, N. Deeb-Sossa, L. García and S. G. García (2012). "Mexico City’s Legal Abortion Program: health workers’ experiences." Revista Panamericana de Salud Publica **32**(6): 399-404.

Ganatra, B. and S. Hirve (2002). "Induced Abortions Among Adolescent Women in Rural Maharashtra, India." Reproductive Health Matters **10**(19): 76-85.

Gober, P. (1997). "The role of access in explaining state abortion rates." Social Science & Medicine **44**(7): 1003-1016.

Henshaw, S. K. (1995). "Factors hindering access to abortion services." Family Planning Perspectives **27**(2): 54-87.

Marlow, H. M., S. Wamugi, E. Yegon, T. Fetters, L. Wanaswa and S. Msipa-Ndebele (2014). "Women’s perceptions about abortion in their communities: perspectives from western Kenya." Reproductive Health Matters **22**(43): 149-158.

Upadhyay, U. D., N. E. Johns, K. R. Meckstroth and J. L. Kerns (2017). "Distance Traveled for an Abortion and Source of Care After Abortion." Obstetrics & Gynecology **130**(3): 616-624.
