# Supplementary material for: The economics of abortion and its links with stigma: A secondary analysis from a scoping review on the economics of abortion
Source: PLoS One. 2021 Feb 18;16(2):e0246238. doi: 10.1371/journal.pone.0246238 (PMC7891754; doi:10.1371/journal.pone.0246238)
Supplement: S8 Appendix — (DOCX) [file pone.0246238.s008.docx]

**S8 Appendix.** **Summary of included studies reporting abortion-related stigma and economic impact at the mesoeconomic level (n=4)**

| **Author, year [country]** | **Aim/objective(s)** | **Population** | **Study type** | **Summary of main findings** |
| --- | --- | --- | --- | --- |
| (Contreras, van Dijk et al. 2011) [Mexico] | To examine the experiences and opinions of health care professionals after the legalization of abortion in Mexico City in 2007 | 64 semi-structured interviews with obstetricians/gynaecologists, nurses, social workers, key decision makers at the Ministry of Health, and others | Qualitative study using semi-structured interviews | The number of conscientious objections was problematic for hospitals and health centres providing legal abortions; reports noted that these objections were not for moral or ethical reasons but rather to avoid extra workloads. Objectors also reported providing abortions in their private practices, suggesting financial incentives. Objecting professionals created hostile environments for care seekers, including making them wait longer for their services. |
| (Foster, LaRoche et al. 2017) [Canada] | To document women's experiences obtaining abortion care in New Brunswick (NB) before and after the Regulation 84-20 amendment; identify the economic and personal costs associated with obtaining abortion care; and examine the ways in which geography, age and language-minority status condition access to care. | New Brunswick residents who received abortion services (n=36). | Qualitative: semi-structured interviews | One study participant explained: I went to my doctor and my doctor at the time flat out said [he was] not willing to help me in any way because it was not [his] beliefs...I've heard of other people…they can't get referrals from doctors to get it done in a regular hospital setting, where Medicare would pay for it because of the doctor's personal beliefs. |
| (Gerdts, DeZordo et al. 2016) [United Kingdom] | To better understand the experiences of non-resident women who travel to the United Kingdom (UK) seeking abortion services. | Non-UK residents seeking abortions at three British Pregnancy Advisory Service (BPAS) clinics (n=58). | Cross-sectional survey | Four women in this study reported not seeking abortion in their country of residence due to clinician refusal to provide abortion. Some participants in this study were forced to travel because they faced procedural barriers to legal abortion care, in particular conscientious objection, in their country of residence. How refusals to provide abortion care are defined and understood by providers, how they are communicated to women, and how such refusals shape women’s decisions to travel requires further exploration. |
| (White, Adams et al. 2019) [United States] | To compare pregnancy options counseling and referral practices at state- and Title X-funded family planning organizations in Texas after enforcement of a policy restricting abortion referrals for providers participating in state-funded programs, which differed from Title X guidelines to provide referrals for services upon request. | Publicly funded family planning organizations in Texas. | Qualitative: semi-structured interviews | Despite none of the study respondents expressing personal views opposing abortion, their perceptions of anti-abortion sentiment in the community may have contributed to staff reluctance to provide women with more information about available services. Some respondents were concerned that providing any information about abortion beyond the name of a provider would threaten their state family planning funding. Therefore, despite examples of permissible practices in the proposed Title X guidelines, providers may censor themselves from offering any abortion-related information. |

Contreras, X., M. G. van Dijk, T. Sanchez and P. S. Smith (2011). "Experiences and Opinions of Health-Care Professionals Regarding Legal Abortion in Mexico City: A Qualitative Study." Studies in Family Planning **42**(3): 183-190.

Foster, A. M., K. J. LaRoche, J. El-Haddad, L. DeGroot and I. M. El-Mowafi (2017). ""If I ever did have a daughter, I wouldn't raise her in New Brunswick:" exploring women's experiences obtaining abortion care before and after policy reform." Contraception **95**(5): 477-484.

Gerdts, C., S. DeZordo, J. Mishtal, J. Barr-Walker and P. A. Lohr (2016). "Experiences of women who travel to England for abortions: an exploratory pilot study." European Journal of Contraception & Reproductive Health Care **21**(5): 401-407.

White, K., K. Adams and K. Hopkins (2019). "Counseling and referrals for women with unplanned pregnancies at publicly funded family planning organizations in Texas." Contraception **99**(1): 48-51.
