# Supplementary material for: The economics of abortion and its links with stigma: A secondary analysis from a scoping review on the economics of abortion
Source: PLoS One. 2021 Feb 18;16(2):e0246238. doi: 10.1371/journal.pone.0246238 (PMC7891754; doi:10.1371/journal.pone.0246238)
Supplement: S9 Appendix — (DOCX) [file pone.0246238.s009.docx]

**S9 Appendix.** **Summary of included studies reporting abortion-related stigma and economic costs at the macroeconomic level (n=4)**

| **Author, year [country]** | **Aim/objective(s)** | **Population** | **Study type** | **Summary of main findings** |
| --- | --- | --- | --- | --- |
| (Aiken, Johnson et al. 2018) [Ireland] | (1) to examine the factors affecting whether women in Ireland choose to access abortion by travelling or by using online telemedicine; and (2) to explore their experiences in accessing care through each pathway | Women (n=38) identified through three organisations: Women on Web, Abortion Support Network, For Reproductive Rights Against Oppression, Sexism and Austerity.  Criteria: aged over 18, had an abortion within 8 years of study, lived in Ireland at time of abortion, had travelled or used telemedicine to access abortion care. | Qualitative in-depth interviews | This study found that the restricted legal environment as well as the concern of judgement in communities led people in Ireland to travel for their abortions. |
| (Chełstowska 2011) [Poland] | To describe the economic consequences of the stigmatisation and illegality of abortion and its almost complete removal from public health services in Poland since the late 1980s. | Polish women | Review | The situation in Poland seems to be similar in Ireland, where regulations regarding abortion are even more restrictive, and the monopolisation of abortion services by the private sector causes social inequality because commercialised abortion services are not affordable for everyone. However, this can be bypassed if one has the means to travel abroad. Women from the middle class, who are more likely to have an influence on the political situation, find these conditions bearable, while women from the working class lack the political capital to protest against the discrimination against them. The effort to change the law cannot succeed unless women from all social classes show solidarity.” |
| (Gerber Fried 1997) [United States] | This paper gives a picture of the status of legal abortion from the vantage point of those women who bear the brunt of restricted access | Low-income women, women of colour (who comprise a disproportionate number of the poor), and young women | Review | Restricting access for young women and denying public funding for abortions are strategic ways to isolate and stigmatize the doctors who perform abortions, the hospitals and clinics where abortions are provided, abortion itself and ultimately, the women who have abortions. Adherents to the anti-abortion movement’s agenda have made moral disapproval a key strategy in their efforts to restrict and re-criminalise abortion and have successfully used it as a vehicle for shaping Public opinion. For example, in the area of public funding, they have made appeals such as: ‘Abortion may be legal, but why should we be forced to pay for something that is morally repugnant (to us)?’. The same contempt for women, especially those who have the fewest resources, pervades other aspects of the conservative political agenda, which calls for the restoration of the stigma of illegitimacy and a renewed emphasis on the connection between illegitimacy, poverty and social decay. |
| (Gober 1997) [United States] | Investigate the role of access in explaining the variation in state abortion rates | U.S. state policies and abortion rates | Regression analysis | The decision to terminate a pregnancy has been transformed from a private matter between a woman and her doctor into a more public decision, subject to public opinion, local discretion over the prosecution of antiabortion violence, local influence over hospital management, and public laws regulating abortion. These public realms vary from place to place. They define how easy, expensive, safe, and socially acceptable it is to obtain an abortion. They determine whether a young woman can obtain an abortion in a nearby facility, free of charge, and free from intimidation or whether she is required to obtain permission from a parent, travel hundreds of miles to the nearest clinic, wait 24 hr alter signing an informed consent form, pay $300, and then face demonstrators as she enters the abortion facility. |

Aiken, A. R. A., D. M. Johnson, K. Broussard and E. Padron (2018). "Experiences of women in Ireland who accessed abortion by travelling abroad or by using abortion medication at home: a qualitative study." BMJ Sex Reprod Health.

Chełstowska, A. (2011). "Stigmatisation and commercialisation of abortion services in Poland: turning sin into gold." Reproductive Health Matters **19**(37): 98-106.

Gerber Fried, M. (1997). "Abortion in the US: Barriers to access." Reproductive Health Matters **5**(9): 37-45.

Gober, P. (1997). "The role of access in explaining state abortion rates." Social Science & Medicine **44**(7): 1003-1016.
