# Supplementary material for: The economics of abortion and its links with stigma: A secondary analysis from a scoping review on the economics of abortion
Source: PLoS One. 2021 Feb 18;16(2):e0246238. doi: 10.1371/journal.pone.0246238 (PMC7891754; doi:10.1371/journal.pone.0246238)
Supplement: S10 Appendix — (DOCX) [file pone.0246238.s010.docx]

**S10 Appendix.** **Summary of included studies reporting abortion-related stigma and economic impact at the macroeconomic level (n=3)**

| **Author, year [country]** | **Aim/objective(s)** | **Population** | **Study type** | **Summary of main findings** |
| --- | --- | --- | --- | --- |
| (Chełstowska 2011) [Poland] | To describe the economic consequences of the stigmatisation and illegality of abortion and its almost complete removal from public health services in Poland since the late 1980s. | Polish women | Review | Stigmatisation of pregnancy termination enforces and obscures, at the same time, the commercialisation and privatisation of abortion in the postsocialist Polish state. The 1993 abortion law was only a part of that process, and a wider policy of limiting access to legal abortion to the minimum. Stigmatisation of abortion was the primary reason why it disappeared from public health services and for the emergence of a market for private abortion services. It is the interpretation and the political intent that pushed legal abortions out of public hospitals and more widely, the public sphere. The monopolisation of abortion services by the private sector causes social inequality because commercialised abortion services are not affordable for everyone. But because stigmatisation of abortion dominates the public discourse, the economic aspects are rarely discussed. Thus, the combined forces of right-wing Catholic ideology and neoliberal economic reforms have resulted in reproductive and social injustice. |
| (Crane and Dusenberry 2004) [Global] | To examine the effect of the Global Gag Rule on family planning organizations in countries receiving American assistance | Family planning organizations within multiple countries | Literature review | The result of this Global Gag Rule has been to institutionalise the stigmatisation of abortion in US foreign assistance for the last three decades. |
| (Felkey and Lybecker 2014) [United States] | The analysis seeks to measure whether young women really are less careful in using contraception if abortions are less costly, both in the context of ﬁnancial and opportunity costs, and explore the impact of direct and indirect abortion restrictions, | Data on women under the age of 25 seeking abortions. | Regression analysis | The variable controlling for a state’s pro-life sentiment was always negative and was signiﬁcant for the youngest women in 2002 and both age groups in 1989. This indicates that states presenting an overall pro-life sentiment inhibit the use of oral contraceptives. In essence, the political climate and social stigma may make it harder for a woman to make the choice to use oral contraceptives. While not a cost in terms of money or time, social stigma increases the cost of oral contraceptives. A reduction of this and other costs may increase the use of oral contraception and reduce unwanted pregnancies. |

Chełstowska, A. (2011). "Stigmatisation and commercialisation of abortion services in Poland: turning sin into gold." Reproductive Health Matters **19**(37): 98-106.

Crane, B. B. and J. Dusenberry (2004). "Power and Politics in International Funding for Reproductive Health: the US Global Gag Rule." Reproductive Health Matters **12**(24): 128-137.

Felkey, A. J. and K. M. Lybecker (2014). "Utilization of oral contraception: The impact of direct and indirect restrictions on access to abortion." The Social Science Journal **51**(1): 44-56.
