# Supplementary material for: The economics of abortion and its links with stigma: A secondary analysis from a scoping review on the economics of abortion
Source: PLoS One. 2021 Feb 18;16(2):e0246238. doi: 10.1371/journal.pone.0246238 (PMC7891754; doi:10.1371/journal.pone.0246238)
Supplement: S1 Table — (DOCX) [file pone.0246238.s011.docx]

**S1 Table. Included studies by region and country**

| *Region/country* | *# of studies* |  | *Region/country* | *# of studies* |
| --- | --- | --- | --- | --- |
| **Northern America** | **10** |  | **Europe** | **4** |
| United States | 9 |  | United Kingdom | 2 |
| Canada | 1 |  | Poland | 1 |
|  |  |  | Ireland | 1 |
|  |  |  |  |  |
| **Africa** | **5** |  | **Latin America & Caribbean** | **4** |
| Cote d’Ivoire | 1 |  | Colombia | 2 |
| Zambia | 2 |  | Mexico | 2 |
| Kenya | 1 |  |  |  |
| Sub-Saharan Africa (regional) | 1 |  |  |  |
|  |  |  | **Oceana** | **1** |
| **Asia** | **6** |  | Australia | 1 |
| India | 1 |  |  |  |
| Indonesia | 1 |  | **Cross-Regional Studies** | **2** |
| Cambodia | 1 |  | Global | 2 |
| Bangladesh | 1 |  |  |  |
| Hong Kong | 1 |  |  |  |
| Nepal | 1 |  |  |  |
|  |  |  |  |  |
|  |  |  | **Total** | **32** |

*Note: Each data point represents the number of included studies covering the specified country.*
