# Supplementary material for: The economics of abortion and its links with stigma: A secondary analysis from a scoping review on the economics of abortion
Source: PLoS One. 2021 Feb 18;16(2):e0246238. doi: 10.1371/journal.pone.0246238 (PMC7891754; doi:10.1371/journal.pone.0246238)
Supplement: S2 Table — (DOCX) [file pone.0246238.s012.docx]

**S2 Table. Characteristics of included studies**

|  | *No. Studies* | *Percent* |
| --- | --- | --- |
| **Type of Data** |  |  |
| Quantitative | 4 | 12.5 |
| Qualitative | 16 | 48.5 |
| Both | 12 | 36.4 |
| **Methodology** |  |  |
| Cohort (before & after) | 1 | 3.0 |
| Qualitative | 15 | 469 |
| Mixed methods | 6 | 18.8 |
| Regression | 1 | 3.1 |
| Review paper | 3 | 9.4 |
| Other | 6 | 18.8 |
| **Country Income Group** |  |  |
| Low | 2 | 6.3 |
| Lower-middle | 8 | 25.0 |
| Upper-middle | 4 | 12.5 |
| High | 16 | 50.0 |
| Multiple | 2 | 6.3 |
| **Geographical Level** |  |  |
| National | 6 | 18.8 |
| Sub-national (e.g. state, city) | 18 | 56.3 |
| Health facility | 3 | 9.4 |
| Other | 5 | 15.6 |
| **Study Population** |  |  |
| National | 2 | 6.3 |
| Geographical location (e.g. urban/rural, region, facility) | 7 | 21.9 |
| Age (e.g. adolescents) | 1 | 3.1 |
| Status as abortion seeker | 7 | 21.9 |
| Multiple answers from list | 10 | 31.3 |
| Other, specify | 1 | 3.1 |
| Abortion provider | 4 | 12.5 |
