# Supplementary material for: The economics of abortion and its links with stigma: A secondary analysis from a scoping review on the economics of abortion
Source: PLoS One. 2021 Feb 18;16(2):e0246238. doi: 10.1371/journal.pone.0246238 (PMC7891754; doi:10.1371/journal.pone.0246238)
Supplement: S3 Table — (DOCX) [file pone.0246238.s013.docx]

#### **S3 Table. Economic content and level of extracted stigma studies**

| **Studies with economic outcomes by level of analysis** | | | | | | |
| --- | --- | --- | --- | --- | --- | --- |
| *Micro-level* | | *Meso-level* | | *Macro-level* | | |
| Costs | 15 | Costs | 10 | Costs | 4 | |
| Econ Impact | 4 | Econ Impact | 4 | Econ Impact | 3 | |
| Benefit/value | 1 | Benefit/value | 0 | Benefit/value | 0 | |
| Total | 20 | Total | 14 | Total | 7 | |
| **Studies on economic cost** | |  | |  | | |
| *Study reported on outcome (cost)* | | *# of studies by level* | | *Total extractions by level* | | |
| Yes | 24 | Micro | 12 | Micro | 15 | |
| No | 8 | Meso | 6 | Meso | 10 | |
| Total | 32 | Macro | 1 | Macro | 4 | |
|  |  | More than one | 5 | Total | 29 | |
|  |  | Total | 24 |  |  | |
| **Studies on economic impact** | | | |  | | |
| *Study reported on outcome (impact)* | | *# of studies by level* | | *Total extractions by level* | | |
| Yes | 11 | Micro | 4 | Micro | 4 | |
| No | 21 | Meso | 4 | Meso | 4 | |
| Total | 32 | Macro | 3 | Macro | 3 | |
|  |  | More than one | 0 | Total | 11 | |
|  |  | Total | 11 |  |  | |
| **Studies on economic benefit/value** | | | | | |  |
| *Study reported on outcome (benefit/value)* | | *# of studies by level* | | *Total extractions by level* | |  |
| Yes | 1 | Micro | 1 | Micro | 1 |  |
| No | 31 | Meso | 0 | Meso | 0 |  |
| Total | 32 | Macro | 0 | Macro | 0 |  |
|  |  | All | 0 | Total | 1 |  |
|  |  | Total | 1 |  |  |  |

Note: Total data extractions by level include data extractions for studies with multiple levels
